# Biochar amendment improves degraded pasturelands in Brazil: environmental and cost-benefit analysis

**DOI:** 10.1038/s41598-019-47647-x

**Published:** 2019-08-19

**Authors:** Agnieszka E. Latawiec, Bernardo B. N. Strassburg, André B. Junqueira, Ednaldo Araujo, Luiz Fernando D. de Moraes, Helena A. N. Pinto, Ana Castro, Marcio Rangel, Gustavo A. Malaguti, Aline F. Rodrigues, Luis Gustavo Barioni, Etelvino H. Novotny, Gerard Cornelissen, Maiara Mendes, Nilcileny Batista, Jose Guilherme Guerra, Everaldo Zonta, Catarina Jakovac, Sarah E. Hale

**Affiliations:** 1 Department of Geography and the Environment, Rio Conservation and Sustainability Science Centre, Pontifical Catholic University of Rio de Janeiro, 22453900 Rio de Janeiro, Brazil; 2 International Institute for Sustainability, Estrada Dona Castorina 124, 22460-320 Rio de Janeiro, Brazil; 3 National School of Tropical Botany (ENBT), Rua Pacheco Leão, 2040 - Solar da Imperatriz, Horto, 22460-036 Rio de Janeiro, Brazil; 4 Institute of Agricultural Engineering and Informatics, Faculty of Production and Power Engineering, University of Agriculture in Kraków, Balicka 116B, 30-149 Kraków, Poland; 5 University of East Anglia, Norwich Research Park, Norwich, NR4 7TJ UK; 6 Federal University of Rio de Janeiro, 68020 Rio de Janeiro, Brazil; 7 Brazilian Agricultural Research Corporation, Embrapa Agrobiology, Rodovia BR 465, Km 7, 23891-000, Seropédica, Rio de Janeiro Brazil Embrapa Agrobiology, Rio de Janeiro, Brazil; 8 Federal Rural University of Rio de Janeiro (UFRRJ), Rodovia BR 465, Km 07, 23890-000 Seropédica, Rio de Janeiro Brazil; 9 Brazilian Agricultural Research Corporation, Embrapa Agricultural Informatics, Av. Dr. André Tosello, 209 - Cidade Universitária, 13083-886 Campinas, São Paulo Brazil; 10 Brazilian Agricultural Research Corporation, Embrapa Soils, R. Jardim Botânico, 1024 - Jardim Botânico, 22460-000 Rio de Janeiro, RJ Brazil; 11 Department of Environmental Engineering, Norwegian Geotechnical Institute, P.O. Box 3930, Ullevål Stadion, N-0806 Oslo, Norway; 12 Institut de Ciència i Tecnologia Ambientals, Universitat Autònoma de Barcelona, 08193 Bellatera, Barcelona Spain

**Keywords:** Environmental impact, Carbon cycle

## Abstract

Most deforested lands in Brazil are occupied by low-productivity cattle ranching. Brazil is the second biggest meat producer worldwide and is projected to increase its agricultural output more than any other country. Biochar has been shown to improve soil properties and agricultural productivity when added to degraded soils, but these effects are context-dependent. The impact of biochar, fertilizer and inoculant on the productivity of forage grasses in Brazil (*Brachiaria* spp. and *Panicum* spp.) was investigated from environmental and socio-economic perspectives. We showed a 27% average increase in *Brachiaria* production over two years but no significant effects of amendment on *Panicum* yield. Biochar addition also increased the contents of macronutrients, soil pH and CEC. Each hectare amended with biochar saved 91 tonnes of CO_2_eq through land sparing effect, 13 tonnes of CO_2_eq sequestered in the soil, equating to U$455 in carbon payments. The costs of biochar production for smallholder farmers, mostly because of labour cost, outweighed the potential benefits of its use. Biochar is 617% more expensive than common fertilizers. Biochar could improve productivity of degraded pasturelands in Brazil if investments in efficient biochar production techniques are used and biochar is subsidized by low emission incentive schemes.

## Introduction

Land covered with forage grasses for animal grazing occupies 26% of global ice-free land^1^ and livestock provides employment and sustenance to nearly one third of the world’s population. Pasturelands contribute significantly (40%) to global agriculture gross domestic product^2^. Inadequate management of pastures and soil degradation throughout the tropics renders pasture productivity below its potential and lead to adverse economic and environmental impacts^3,4^. In Brazil, the country that holds the world’s largest commercial cattle herd^1,5^, pasture-fed cattle ranching occupies 158 million hectares corresponding to 75% of the country’s agricultural land^3^. Brazilian cattle ranching is characterized by low stocking rates, mostly below 1 animal unit (AU) per hectare (1 AU = 0.7 animal), a rather low number comparing with other meat-producing countries^3^. Labour cost, shortage of qualified technical extension assistants and high costs to maintain good quality of soils are among principal reasons for low productivity of Brazilian cattle ranching^3,5–7^. Overgrazing, erosion and land availability that historically hurdled application of good agricultural practices contributed to the prevalence of land degradation in Brazil^5^. More than 70% of pastures in Brazil are classified as degraded^8^.

Degraded Brazilian pasturelands have impacts beyond the country level. Together with agriculture, they are linked to deforestation and biodiversity loss at an unprecedented scale, and greenhouse gases emissions of global significance^9^. If projections are realized, Brazil will see the highest worldwide increase in meat production in the following decade^10^. To avoid negative impacts of expanding cattle ranching, improving productivity of already converted lands has been proposed as a key solution to conciliate development with conservation^11^. In Brazil, pastureland productivity could be tripled in much more sustainable ways^3^, providing meat and other commodities while reversing environmental degradation. This is particularly important for smallholders who represent the majority (70%) of cattle ranchers in Brazil and often strive to maintain profits. In addition, the Brazilian Native Vegetation Protection Law and governmental commitments^12^ oblige producers to spare land for conservation. This may generate competition for land as many producers strive to continue with low-productivity cattle ranching to meet the ever-increasing national and international demands for meat, while simultaneously seeking to keep a part of their land covered with native vegetation. Legally imposed restoration is projected to occur throughout Brazilian biomes with the Atlantic Rainforest biome expected to restore the largest areas^13,14^ (Supplementary Fig. S1). The Atlantic Rainforest biome is a global biodiversity hotspot, concentrating 95% of the Brazilian population and 80% of the national Gross Domestic Product.

Pasture degradation in the Atlantic Forest biome has a range of direct negative impacts on the provision of ecosystem services, such as water and food production, and carbon sequestration. Despite being a fundamental resource for human well-being and being increasingly recognised in environmental policies^15^, soil ecosystem services remain poorly understood, overlooked and largely excluded from the studies on ecosystem services valuation^16^. Loss of soil carbon is at the heart of land degradation; both in terms of increasing carbon dioxide emissions to the atmosphere and of the damage it does to soil physical, chemical and biological attributes. Including the valuation of soil ecosystem services into decision making is not only paramount for minimizing carbon emissions, but also for improving local livelihoods and promoting food security.

Biochar (a carbon-rich product resulting from the pyrolysis of organic residues) has emerged as a potential solution to restore soils, increase agricultural performance and sequester carbon^17^. Biochar was shown to improve soil pH, nutrient content and water holding capacity^18^; applied alone or combined with limestone or inoculant^19,20^. Biochar is often accompanied by ash that is rich in macronutrients such as Ca, Mg, K, important for soil health^21^. Slavich *et al*. (2013)^22^ showed that biochar from feedlot manure increased pasture productivity by 11% and improved the agronomic nitrogen use efficiency by 23%. A meta-analysis has been carried out based on 128 observations of biochar decomposition in soil. A mean residence time of labile and recalcitrant biochar carbon pools were reported to be 108 days and 556 years respectively. Three percent of the biochar carbon was contained in the labile pool. Biochar was also shown to retard the mineralisation of soil organic matter and stimulate microbial activity^23^. Studies that investigate the potential of biochar to improve forage grass productivity and diminish adverse impacts of cattle ranching are however scarce.

This study investigates biochar amended to Brazilian pasturelands with the aim of increasing yield of the two most common forage grasses in Brazil; *Brachiaria brizantha* cv. Marandu and *Panicum maximum* cv. Mombaça. Biochar may present an interesting alternative in pasture management in Brazil as farmers use charred organic residues (so called ‘moinha’) to increase soil carbon, nutrients and correct pH. It is also common in rural areas of Brazil to burn organic residues. Introducing simple stoves for biochar production from these residues could reduce risks related uncontrolled fire spread and health issues (Supplementary Video S1). Pot trials in controlled conditions for six harvest cycles, a field study over two harvest cycles, and a socio-economic survey were carried out and provided the most comprehensive up-to-date analysis of biochar use in Brazilian pasturelands. This study has three main aims: (1) investigate the impacts of biochar on forage yield and soil properties; (2) perform a socio-economic analysis of biochar use in small-holder organic farming; (3) valuation of ecosystem services related to food production and carbon sequestration that result from biochar amendment to soil (case study for the state of Rio de Janeiro). To the best of our knowledge this is the first study that investigates the use of biochar in Brazilian pastures, one of the very few worldwide studies on biochar amendment to pastureland, and the first study to report a socio-economic analysis of biochar amendment to improve sustainability of cattle ranching.

## Methods

### Study site

The pot experiment was performed in a greenhouse (22.97′S, 43.24′W) where the temperature was recorded daily and watering frequency and dose were adjusted to reflect field conditions. The field experiment was performed at the Brazilian Agricultural Research Corporation (Embrapa Agrobiologia - 22°45′S; 43°41′W). Pot experiments and field trials were carried out in Rio de Janeiro, Brazil. Additional site information is included in Supplementary methods and Supplementary Fig. S2. Experiments were conducted on soil classified as Planosol^24^.

### Experimental design and pasture management

Tested forage grasses were *Brachiaria* and *Panicum*. A full factorial design was delineated (2^3^ for *Brachiaria* and 2^2^ for *Panicum*) with or without: inoculant (*Azospirillum brasilense* strain sp. 245; used only for *Brachiaria*); fertilizer (562 kg termophosphate per hectare with 18% of P_2_O_5_ (100 kg/ha) and potassium sulphate with 50% of K_2_O (120 kg/ha); and biochar (15 t/ha). Control did not include amendments. Inoculum (50 g for each 10 kg of seeds) was produced with turf and contained 108 colony-forming units/g. A 10% sucrose solution was used as an adherent. In the pot experiment, grasses were originally sown on soil collected from field experiment at a density of 10 seeds/pot for *Brachiaria* and 12 seeds/pot for *Panicum* in 10 L pots, as recommended by the seed provider. Posteriorly, four plants were maintained per pot in order to standardize the experiment. Biochar, fertilizer and/or inoculant were mixed at the top 10 cm of the pots. Each treatment was replicated five times for each grass and aboveground biomass was harvested and measured six times during 15 months (68, 139, 207, 269, 361 and 454 days after sowing, starting February 2015). In the field, 40 plots (2 m × 2 m each) were established in May 2014 in a randomized block experimental design. *Brachiaria* was sowed at a density of 20 seeds/plot and *Panicum* at 55 seeds/plot. Initially, plots were homogenously irrigated to facilitate pasture establishment and prevent possible germination problems due to a severe drought at the beginning of the experiment. Biochar, fertilizer and/or inoculant were spread on soil surface and incorporated manually into soil to approximately 10 cm of depth. Each treatment was replicated three times for *Brachiaria* and four times for *Panicum*, and the biomass was harvested twice for *Brachiaria* (95 and 170 days after sowing) and three times for *Panicum* (95, 143 and 214 days after sowing).

### Biochar production

In Seropédica, the species *Gliricidia sepium* (family Fabaceae) is invasive and must be systematically cut, providing large amounts of organic residues. Gliricidia has a high biomass production capacity in various tropical conditions up to 800 m altitude. Being a perennial and easily cultivated plant for green manuring (N fixation) and mulching, Gliricidia’s thicker stalks can be used for biochar production as they do not have alternative use and may cause undesired shadow. Gliricidia tolerates frequent pruning of about four times per year. Therefore, this biomass feedstock was chosen for biochar production and can be transferrable to other tropical conditions. Traditional brick kiln^25^ was used, since it presents desirable-quality biochar. The analysis of physicaland chemical biochar properties (Supplementary Fig. S3) followed the methods described in Martinsen *et al*.^26^.

### Biomass analysis

In the pot experiment and field trial, the analysis of grass aboveground biomass included measuring wet and dry weights as well as macro and micronutrients. Aboveground biomass was weighed after harvest (leaving 5 cm of forage for regrowth) for wet weight, and again after drying in the oven for 96 hours (at 60 °C) for dry weight. After each harvest, a leaf sample of about 200 g was collected, from which the concentration of macronutrients (P, K, Ca, Mg and S, in g/kg) and micronutrients (Cu, Fe, Mn and Zn, in mg/kg) were measured following the protocol of Malavota *et al*.^27^. Sulfuric acid digestion was used to analyze N. Roots were analyzed at the end of the pot experiments (Supplementary methods).

### Soil sampling and analysis

For pot experiments, soil samples were collected and analyzed at the beginning and at the end of the experiment (February 6, 2015 and May 5, 2016). In the field trial, soil samples were collected at the end of each harvest cycle (dates in Supplementary Methods). Five subsamples along two diagonal lines through each plot were collected and pooled into one composite sample. Soil samples were homogenized and sieved to 2 mm, and analyzed for pH (H_2_O, KCl and CaCl_2_), plant-available water (difference between water retention at 0.10 and 15 atm, i.e., pF 2 and 4.2, respectively), organic matter (dag/kg), total N (g/kg), total K (mg/dm^3^), total P (mg/dm^3^), P residual (mg/L), total Mg (cmol/dm^3^), H + Al (cmol/dm^3^), Na (mg/dm^3^), Ca (cmol/dm^3^), SB (cmol/dm^3^), CEC (effective and potential; cmol/dm^3^), Zn (mg/dm^3^), Fe (mg/dm^3^), v (%), m (%), Mn (mg/dm^3^), Cu (mg/dm^3^) and soil texture. Residual P, organic matter, Zn, Cu, Mn and Fe were determined using Mehlich method. C, H and N percent in the pot experiment was determined using Dumas method via dry combustion of 5.0 mg (±0.1 mg) soil in an element analyzer, PerkinElmer 2400. Acetanilide was used as reference material. For more information check Supplementary Methods.

### Statistical analysis

Aboveground biomass weight data were analyzed using repeated measures linear mixed effect models, using ‘time’ (i.e., the different harvests through time), and the treatments: biochar, fertilizer, inoculant and their interactions as fixed factors and ‘subject’ (i.e., each pot or plot) as a random (blocking) factor. Root biomass weight data were analyzed using generalized linear models, since these data were obtained at a single time (at the end of the pot experiment). For the analyses of multivariate data (biomass nutrient concentration and soil parameters), we first performed Principal Components Analyses (PCAs) on each dataset (after centering and standardizing the variables) and then used the first two axes (or principal components) in further analyses. For the pot experiment, PCA axes were used as dependent variables in either repeated measures linear mixed effect models (for biomass nutrient concentration, given that this data was measured several times through time) or in generalized linear models (for soil parameters, given that this was measured only at the end of the pot experiment), with the same structure as the models described above. For the field trials, PCA axes were used as dependent variables in repeated measures linear mixed effect models for both biomass nutrient concentration and for soil parameters (given these variables were repeatedly measured in time). All analyses were performed in R^28^, using packages *stats*, *lme4*^29^ and *MuMIn*^30^.

### Cost-benefit analysis and ecosystem services valuation

Two ecosystem services were valuated: food provision and carbon sequestration. To calculate food production, biomass from each treatment was compared with the control (Supplementary Methods - Meat profit calculation from biomass). This value was used to calculate the additional US$/ha for each treatment estimated from the additional potential beef production, using Equation (1).

1 $$M={\rm{\Delta }}B\times r\times f\times p,$$

in which:

M = additional profit meat

∆B = difference of the biomass generated with the treatment in relation to the control;

r = 0.026 = value of the equivalent ton of carcass per ton of dry mass ingested, in a modal system with a complete cycle of meat production for the Atlantic Forest, with an efficiency rate of 100%^31^

f = forage productivity, value of minimum or maximum productivity (between 10 and 17 tDM/ha) of the grass Brachiaria cv. Marandu, in tDM/ha^32^;

p = meat price, in US$, the average price of meat in the State of Rio de Janeiro during the experiment period at the producer level was R$9.42/kg^33^ and the exchange rate, R$3.51/US$.

For carbon sequestration, land sparing effects and direct carbon storage in soil adding biochar were evaluated. Land sparing assumes that for each hectare with improved productivity, a proportion of land will be freed up for other uses^3^. In the Atlantic Forest, the carbon saved is 443.7 tCO_2_eq/ha. This was multiplied by land spared by intensification with biochar (27%) and by the unit carbon price (US$5/tCO_2_eq). The value of carbon sequestered in soil was obtained from the weight balance determined in the pot experiment (more information in Supplementary Table S1).

The cost of biochar production in different stoves was estimated from equipment and labour costs through field research (all details in Supplementary Table S2, S3 and S4; Supplementary Methods). According to Brazilian labour law, an employee can work up to 44 hours/week, being 8 hours a day. These values were considered in our research.

The analysis was carried out for scenarios with and without labour costs. Cost of production in each additional kiln was calculated considering that kilns can be operated in parallel. Cost-benefit analysis of biochar, fertilizer and inoculant were calculated. Following costs were taken into account: fertilizer (U$0.3/kg for thermophosphate and U$6/kg for potassium sulphate), transportation (IEA/SP)^34^, inoculant (U$0.45/g) and lime (U$0.02/kg) (other details, Supplementary Methods).

The minimum carbon price was calculated (Supplementary Methods and Supplementary Table S5). Finally, to make biochar economically viable, three different scenarios were considered: (a) biochar in the soil only during the experiment period, (b) biochar reduction, in a linear tendency, until zero at the end of 10 years and (c) the same gain with biochar for 10 years, without any loss. For each scenario, net present value (NPV) was calculated based on meat production gains, using the attractiveness rate of 6%. The resulting values were divided by the amount of carbon sequestered in each hectare.

## Results

### Forage productivity and nutrient content

**Figure 1:**
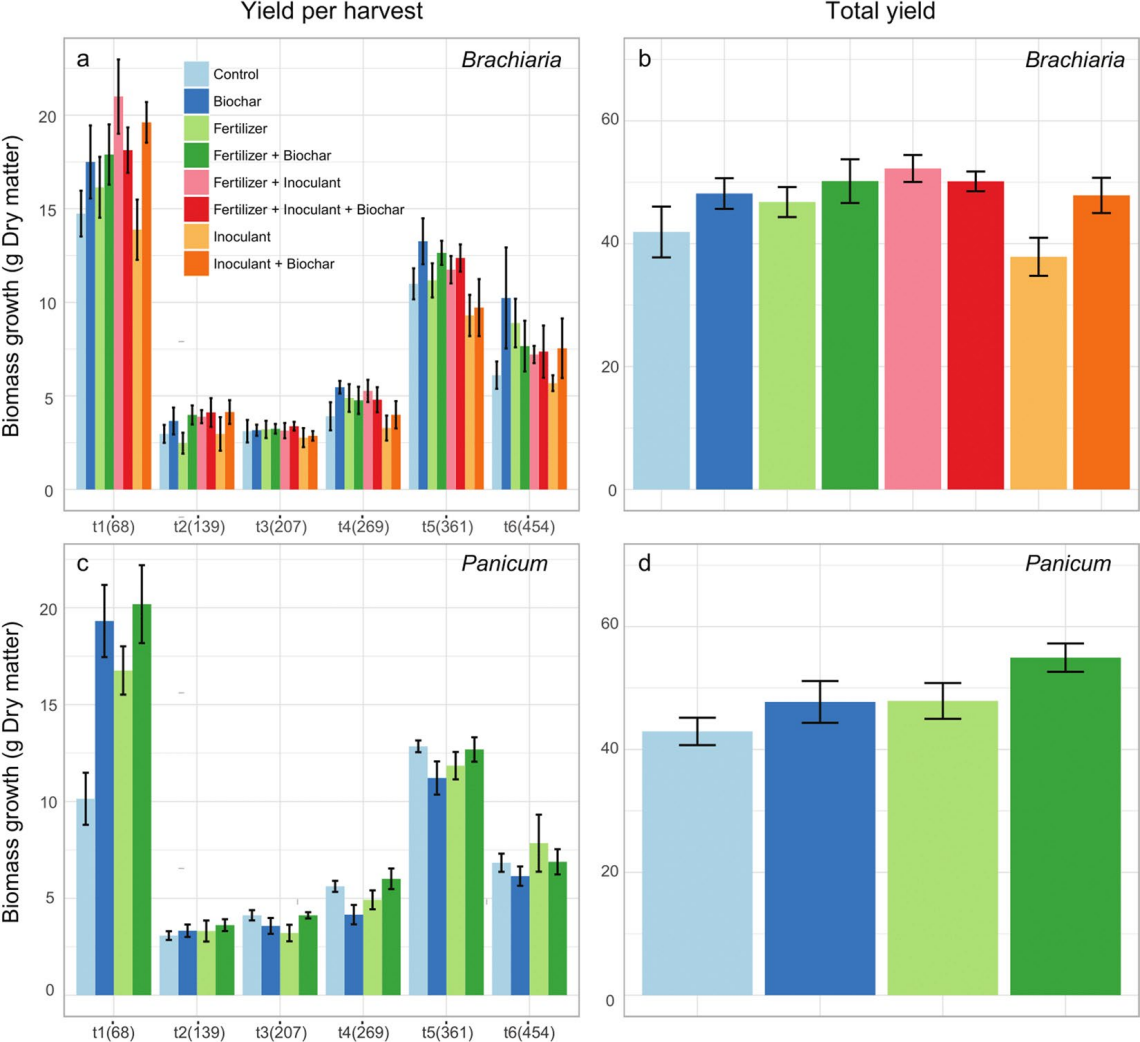
Aboveground biomass was harvested in six different times (68, 139, 207, 269, 361 and 454 days after planting). Left panels (**a**,**c**) show the yield in each of the harvests, and the right panels (**b**,**d**) show the total yield of the experiment. Vertical lines above each bar show the standard error.

**Figure 2:**
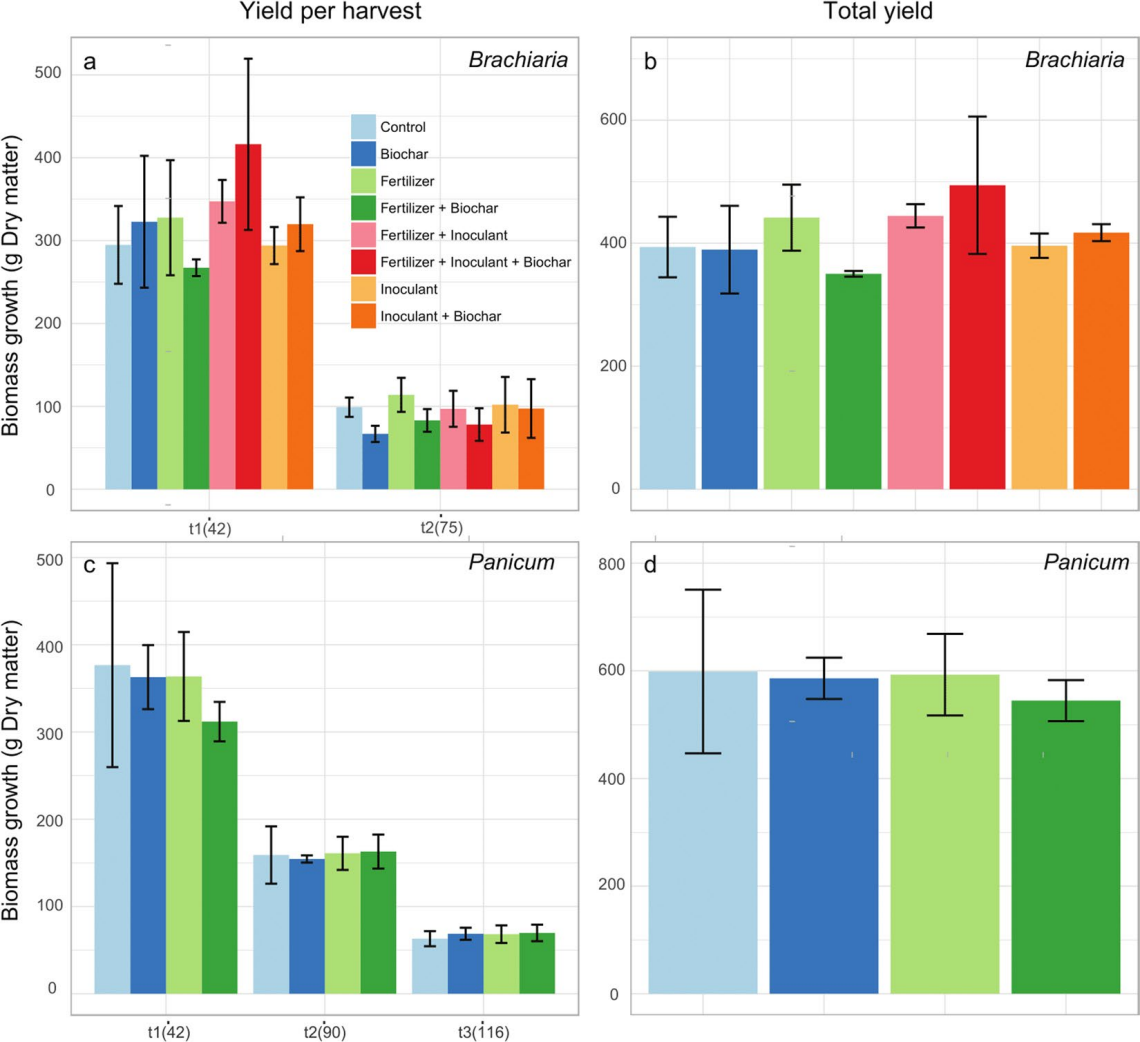
Aboveground biomass was harvested in two or three different times (42 and 75 days after planting for *Brachiaria*, and 42, 90 and 116 after planting for *Panicum*) in the field trial. Left panels (**a**,**c**) show the yield in each of the harvests, and the right panels (**b**,**d**) show the total yield of the trial. Vertical lines above each bar show the standard error.

In the pot experiment, the addition of either biochar or fertilizer led to a significant increase in aboveground biomass of *Brachiaria* in all harvests (both dry and fresh biomass; Fig. 1 and Table 1 and 2; biochar, dry biomass: F = 6.57, p = 0.015; biochar, fresh biomass: F = 11.98, p = 0.002; fertilizer, dry biomass: F = 4.37, p = 0.045; fertilizer, fresh biomass: F = 4.23,p = 0.048). There was a significant interaction between inoculant and time (dry biomass: F = 2.34, p = 0.045; fresh biomass: F = 2.9, p = 0.015), and in general inoculant negatively impacted aboveground biomass production in most harvests (Table 2). For dry aboveground biomass, we also observed a significant interaction between biochar and fertilizer (F = 4.24, p = 0.048), in which the treatment where these two were combined showed an intermediate level of productivity between biochar only (highest) and fertilizer only (lowest; Table 2). For *Panicum*, there was a significant interaction between biochar and time (dry biomass: F = 8.87, p < 0.001; fresh biomass: F = 12.51, p < 0.001), fertilizer and time (dry biomass: F = 2.84,p = 0.021; fresh biomass: F = 4.48, p = 0.001), as well as a third order interaction between these three variables (dry biomass: F = 4.74, p = 0.001; fresh biomass: F = 6.61, p < 0.001; Table 1), indicating a much less consistent and unstable effect of these variables over time on grass productivity. For this forage grass, biochar or fertilizer had strong positive effect in the first harvest, but this effect tended to disappear, and even invert, with time (Fig. 1c,d). Considering the relative differences between treatments and control in aboveground biomass (Table 2), the largest cumulative difference was for biochar for *Brachiaria* (27%) and a combination of biochar with fertilizer for *Panicum* (25.5%). Regarding the root biomass measured at the end of the pot experiment, we found no effects of treatments nor of their interactions on root dry or fresh biomass, for both grasses (Supplementary Table S6). In the field trials, we did not observe statistically significant effects of the treatments on aboveground biomass productivity for both grasses (Fig. 2; Supplementary Tables S7).

**Table 1:** Results of mixed effect models with six repeated measures on the effects of Biochar, Fertilizer and Inoculant (for* Brachiaria* only) (and their interactions) on the dry and fresh biomass of Brachiaria and Panicum forage grasses in a pot experiment. SS – sum of squares; MSS – mean sum of squares; DF – degrees of freedom; R2c – conditional R2; R2m – marginal R2.

Factor	Dry biomass						Fresh biomass					
	SS	MSS	DF	DenDF	F	p	SS	MSS	DF	DenDF	F	p
**Forage grass: Brachiaria**												
Biochar	0.80	0.80	1	31.9	6.57	**0**.**015**	5.31	5.31	1	32.5	11.98	**0**.**002**
Fertilizer	0.54	0.54	1	31.9	4.37	**0**.**045**	1.87	1.87	1	32.5	4.23	**0**.**048**
Inoculant	0.05	0.05	1	31.9	0.42	0.520	0.09	0.09	1	32.5	0.19	0.663
Time	180.30	36.06	5	157.4	294.71	**<2e-16**	457.69	91.54	5	157.9	206.46	**<2**.**2e-16**
Biochar*Fertilizer	0.52	0.52	1	31.9	4.24	**0**.**048**	1.19	1.19	1	32.5	2.68	0.111
Biochar*Inoculant	0.07	0.07	1	31.9	0.59	0.449	0.05	0.05	1	32.5	0.11	0.745
Fertilizer*Inoculant	0.38	0.38	1	31.9	3.13	0.086	0.47	0.47	1	32.5	1.05	0.313
Biochar*Time	0.33	0.07	5	157.4	0.53	0.751	0.55	0.11	5	157.9	0.25	0.941
Fertilizer*Time	0.27	0.05	5	157.4	0.44	0.819	0.70	0.14	5	157.9	0.31	0.904
Inoculant*Time	1.43	0.29	5	157.4	2.34	**0**.**045**	6.44	1.29	5	157.9	2.90	**0**.**015**
Biochar*Fertilizer*Inoculant	0.01	0.01	1	31.9	0.11	0.741	0.19	0.19	1	32.5	0.44	0.513
Biochar*Fertilizer*Time	1.05	0.21	5	157.4	1.71	0.135	1.68	0.34	5	157.9	0.76	0.580
Biochar*Inoculant*Time	0.08	0.02	5	157.4	0.13	0.985	1.09	0.22	5	157.9	0.49	0.783
Fertilizer*Inoculant*Time	0.21	0.04	5	157.4	0.34	0.888	0.79	0.16	5	157.9	0.36	0.878
Biochar*Fertilizer*Inoculant*Time	0.98	0.20	5	157.4	1.60	0.163	2.78	0.56	5	157.9	1.26	0.286
R^2^m	0.84						0.80					
R^2^c	0.87						0.83					
**Forage grass: Panicum**												
Biochar	0.14	0.14	1	16.1	2.10	0.166	0.36	0.36	1	16.0	1.37	0.258
Fertilizer	0.21	0.21	1	16.1	3.11	0.097	0.24	0.24	1	16.0	0.93	0.350
Time	78.69	15.74	5	78.4	234.23	**<2**.**2e-16**	192.58	38.52	5	78.1	147.02	**<2**.**2e-16**
Biochar*Fertilizer	0.02	0.02	1	16.1	0.31	0.583	0.00	0.00	1	16.0	0.01	0.918
Biochar*Time	2.98	0.60	5	78.4	8.87	**0**.**000**	16.38	3.28	5	78.1	12.51	**0**.**000**
Fertilizer*Time	0.95	0.19	5	78.4	2.84	**0**.**021**	5.87	1.18	5	78.1	4.48	**0**.**001**
Biochar*Fertilizer*Time	1.59	0.32	5	78.4	4.74	**0**.**001**	8.66	1.73	5	78.1	6.61	**0**.**000**
R^2^m	0.89						0.8					
R^2^c	0.92						0.89

**Table 2:** Percent differences between treatments and control regarding dry and fresh matter of two forage grasses (*Brachiaria* and *Panicum*) in the pot experiment in a full factorial design. Measurements were taken at six different times (´harvests´). B – biochar; F – fertilizer; I – Inoculant.

	Harvest	Brachiaria							Panicum		
		B	F	B + F	F + I	B + F + I	I	B + I	B	F	B + F
**Dry matter**											
Average Cumulative difference	1	18,7	9,6	21,4	42,4	23,0	−5,8	33,1	90,5	65,3	99,1
	2	23,3	−16,8	34,1	30,9	38,6	−0,1	39,3	8,2	7,7	17,6
	3	1,7	3,0	4,1	1,0	8,4	−11,1	−8,1	−13,3	−22,2	0,0
	4	39,7	25,0	21,8	34,7	22,7	−16,0	2,0	−26,0	−12,4	6,9
	5	20,7	1,7	15,1	6,9	12,6	−15,4	−11,6	−12,7	−7,8	−1,3
	6	67,5	45,5	25,4	18,0	20,6	−7,0	23,5	−10,1	14,7	0,7
		28,6	11,3	20,3	22,3	21,0	−9,2	13,0	6,1	7,6	20,5
		27,4	11,8	20,0	24,9	19,9	−9,5	14,4	11,9	12,3	25,5
**Fresh matter**											
Average Cumulative difference	1	15,4	8,8	19,9	41,9	33,8	−0,7	44,1	128,0	90,2	135,4
	2	19,7	−4,3	24,4	30,9	31,1	−1,8	44,9	7,8	5,0	12,6
	3	11,6	3,6	22,7	−1,1	25,1	−11,6	2,9	−5,1	−15,9	−0,2
	4	44,2	14,2	33,1	14,2	26,9	−13,5	−2,8	−25,1	−26,0	0,1
	5	19,8	15,8	16,7	16,3	27,0	−5,7	39,4	−19,5	−2,2	−15,1
	6	31,8	31,1	18,7	6,5	32,9	−15,1	22,3	−8,6	13,1	5,8
		23,8	11,5	22,6	18,1	29,5	−8,1	25,2	12,9	10,7	23,1
		22,4	10,6	22,4	24,3	30,6	−6,4	28,9	21,9	15,6	32,9

**Figure 3:**
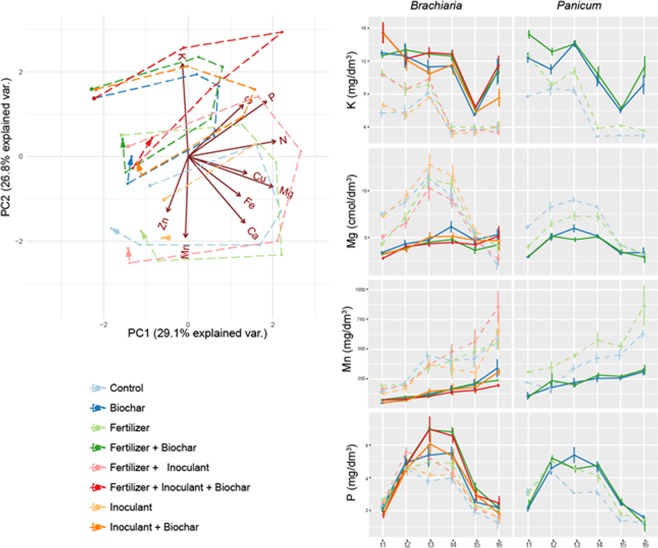
Left panel: Principal Components Analysis of foliar nutrient concentration, measured in a pot experiment with two forage grasses (*Brachiaria* and *Panicum*). Foliar nutrient concentration was measured in six different times (harvests). Each point in the figure represents the centroid of five replicates (pots) measured at a given time, their colors indicate the different treatments and the dashed lines connecting the centroids indicate the changes in nutrient contents through time (harvests), with arrows indicating nutrient contents measured at the last harvest. Vectors indicate the soil variables included in the PCA, and their length and direction indicate the magnitude and direction in which they contribute to the ordination, respectively. Values between brackets indicate the percentage of the variation in the original dataset that is explained by axes PC1 and PC2. Right panel: variation of selected foliar nutrients through time, in the same pot experiment.

Figure 3 presents the results of a Principal Components Analysis (PCA) of biomass nutrient content for the pot experiment for *Brachiaria* and *Panicum*. Loadings of the variables included in the PCA are in Supplementary Table S8. The first axis of the PCA (PC1) explained 29.1% of the variation in the dataset, and was positively correlated mainly with N, P and Mg (Fig. 3; Supplementary Table S8). The second axis (PC2) explained 26.8% of the variation and was correlated positively with K and negatively with Ca, Mn and Zn (Fig. 3, Supplementary Table S8). The effect of biochar amendment is clearly observed on axis PC2 (*Brachiaria:* F = 327.9, p < 0.001; *Panicum:* F = 357.09, p < 0.001), given that the leaves of plants of both grasses growing on biochar-enriched substrates tended to have higher concentrations of K but lower of Mn and Zn (Fig. 3). Biochar had also a significant effect on axis PC1 (*Brachiaria*: F = 49.23, p < 0.001; *Panicum*: F = 70.43, p < 0.001; Supplementary Table S9), because treatments with biochar tended to reach lower concentrations of nutrients such as N, Mg and Ca, which are strongly related to axis PC1. Fertilizer had also a significant effect on axis PC2 (F = 16.94, p < 0.001) (Supplementary Table S9), although only for *Brachiaria*, indicating that the addition of fertilizer tended to increase leaf concentrations of N, P and Mg. The significant interactions between treatments and time (harvest; Supplementary Table S9) resulted from the expected strong temporal variation in leaf nutrient concentration, stemming from the fact that plants absorbed soil nutrients and grew in biomass at different rates through time (Fig. 2). When analyzed individually, most of the nutrients (7 out of 10: N, P, Ca, Mg, Mg, S, Cu, Fe) in the biomass of both grasses followed similar patterns: relatively lower concentrations in the first harvest cycle, increasing over the second, third and fourth cycles, and diminishing towards the end of the experiment. Regarding the analyses of nutrient content in the root biomass in the pot experiment, we did not find any effect of treatments nor of their interactions on the two first axes of a PCA on root nutrient parameters (Supplementary Tables S10 and S11; Supplementary Fig. S4). In the field trials, we found similar patterns for leaf nutrient concentration (although only for *Brachiaria*), in which treatments with biochar tended to have higher leaf concentrations of K but lower of Mn, Mg and Ca (Supplementary Tables S12 and S13; Supplementary Fig. S5).

### Soil properties

**Figure 4:**
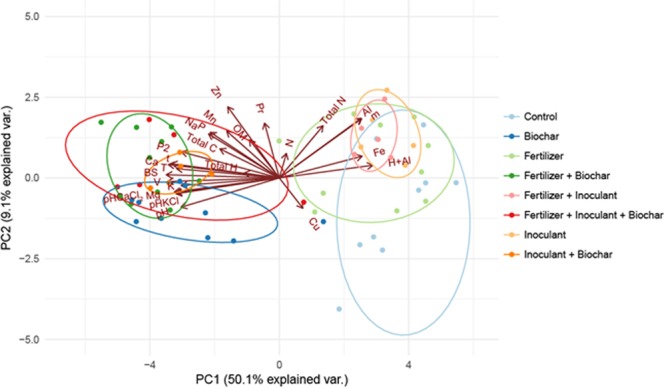
Principal Components Analysis of soil chemical parameters measured in a greenhouse experiment with two forage grasses (*Panicum* and *Brachiaria*). Each point in the figure represents a replicate (pot), and their colors indicate the different treatments. Vectors indicate the soil variables included in the PCA, and their length and direction indicate the magnitude and direction in which they contribute to the ordination, respectively. Values between brackets indicate the percentage of the variation in the original dataset that is explained by axes PC1 and PC2.

Figure 4 presents the results of a PCA on soil parameters performed at the end of the pot experiment for *Brachiaria* and *Panicum*. Loadings of the variables included in the PCA are in Supplementary Table S14. The first axis of the PCA (PC1) explained 50.1% of the variation in the original dataset, and was positively correlated mainly with Fe, Al, H + Al and m and negatively correlated mainly with K, P, Ca, Na, Ca, Mg, base sums, cation exchange capacity and pH (Fig. 3; Supplementary Table S14). The second axis (PC2) explained only 9.1% of the variation and was weakly positively correlated with Zn (Fig. 4, Supplementary Table S14). Biochar had a strong effect on axis PC1 for both grasses (*Brachiaria:* t = −7.74, p < 0.001; *Panicum:* t = −7.48, p < 0.001; Table 3), which is also visible on Fig. 4, because soils on treatments with biochar tended to become less acidic, with increased levels of most macro and micronutrients, and lower levels of Al and Fe. Treatment effects on axis PC2 were weaker and less consistent (as this axis is not strongly correlated with any of the variables and explains little variation in the dataset): for *Brachiaria*, treatments with biochar showed lower values along this axis (t = −2.94, p = 0.006; Table 3), while for *Panicum* treatments with either biochar or fertilizer shower higher values along this axis (biochar: t = 3.26, p = 0.005; fertilizer: t = 3.97, p = 0.001; Table 3). In the field trials, we found similar although weaker effects on soil properties, in which treatments with biochar showed higher levels of Ca, Mg, base sums, t and P-resin (for *Brachiaria*) or higher pH (for *Panicum*; Supplementary Fig. S6; Supplementary Tables S15 and S16).

**Table 3:** Results of generalized linear models showing the effects of biochar, fertilizer and inoculant (and their interactions) on soil chemical parameters measured in a greenhouse experiment with two forage grasses (*Panicum* and *Brachiaria*). PCA1 and PCA2 are the first two axes of a Principal Components Analysis summarizing the variation in soil parameters, explaining 50.1 and 9.1% of the original variation in soil parameters, respectively. Est – estimated coefficient; SE – Standard error of the estimate. Significant p-values are highlighted in bold.

Treatment	Axis PCA1				Axis PCA2			
	Est.	SE	t	p	Est.	SE	t	p
***Forage grass: Brachiaria***								
Intercept	4.351	0.574	7.58	0.000	0.437	0.395	1.11	0.277
Biochar	−6.285	0.812	−7.74	**0**.**000**	−1.640	0.559	−2.94	**0**.**006**
Fertilizer	−0.632	0.812	−0.78	0.442	0.904	0.559	1.62	0.116
Inoculant	−0.997	0.812	−1.23	0.228	0.938	0.559	1.68	0.103
Biochar*Fertilizer	−1.399	1.148	−1.22	0.232	0.365	0.790	0.46	0.647
Biochar*Inoculant	−0.188	1.148	−0.16	0.871	0.426	0.790	0.54	0.593
Fertilizer*Inoculant	0.204	1.148	0.18	0.860	−0.970	0.790	−1.23	0.228
Biochar*Fertilizer*Inoculant	1.781	1.624	1.10	0.281	−0.077	1.117	−0.07	0.945
***Forage grass: Panicum***								
Intercept	3.318	0.651	5.10	0.000	−3.198	0.494	−6.48	0.000
Biochar	−6.882	0.920	−7.48	**0**.**000**	2.278	0.698	3.26	**0**.**005**
Fertilizer	−1.197	0.920	−1.30	0.212	2.774	0.698	3.97	**0**.**001**
Biochar*Fertilizer	0.728	1.301	0.56	0.584	−1.177	0.987	−1.19	0.251

### Cost-benefit analysis and ecosystem services valuation

Based on the average differences in biomass production of each treatment relative to the control, the additional meat production during the experiment period (454 days = 15 months) (t/ha) and additional profits to the farmer were calculated taking into account forage productivity and meat price (Supplementary Table S17).

The highest additional meat production and profit was observed for the treatment with biochar (between US$191 and US$324/ha), followed by the combination of fertilizer with inoculant (between US$172 and US$295/ha) and biochar with fertilizer (between US$139 and US$237; Supplementary Table S17). The treatment with only inoculant had a negative impact on biomass and consequently on profits when compared to the control.

**Figure 5:**
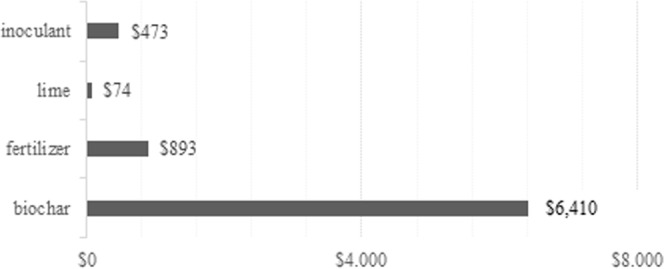
Comparison of the costs, in US$, of each treatment used in the experiment associated with forage grasses *Brachiaria*. For each treatment, represented in each bar of the graph, the cost in dollars was estimated considering that 15t of biochar, 10 kg of inoculant, 3t of limestone and 682.5 kg of fertilizer were used for each experiment with the forage grass, *Brachiaria*.

If the biochar was produced for charcoal or other industrial purposes instead of being amended to the soil, farmers could additionally profit between US$30 and US$85 per month, considering the maximum of number of kilns that can be operated simultaneously without the necessity of additional labour. However, biochar production costs are high when juxtaposed either with increased productivity or profit from direct sale. To prepare enough biochar to apply in one hectare at the doses in our experiment (15t/ha), between 150 and 583 operating stoves (depending on the type) would be necessary and between 75 and 210 people to operate those stoves. At the same time, the profit generated by the sale during the experiment period, in full operation of the stoves, without falling yield and without stove maintenance in that period, the revenues by biochar sale would be lower than the costs, covering only between about 8 to 22% of total costs. Applying to the soil 15 t/ha of biochar costs US$6,410, while 682.5 kg/ha of fertilizer would cost US$ 893, and 10 kg/ha of inoculant would cost US$473 and 3t/ha of lime US$74 (Fig. 5). Given increased productivity following the use of biochar (27%) and fertilizer (12%), fertilizer is 618% less expensive than biochar, assuming just one application of each amendment as performed in the experiment.

Regarding sequestered carbon, each hectare intensified with biochar is associated with US$455 worth of carbon from land sparing effect. Given land intensification targets of the state of Rio de Janeiro (180 000 ha^35^), this may result in 16 383 832 tonnes of CO_2_eq saved with the value of US$ 82 million. If additional sequestration of carbon in soil from biochar use per hectare is considered (13 tonnes CO_2_eq/ha), the final estimates are 18 714 688 CO_2_eq worth US$ 94 million. Although GHG from biochar production were not considered in our study due to practical reasons, other publications report that biochar produced in simple stoves such as those used in our study contribute to minimizing GHG emissions^36^.

We also calculated the NPV based on the data from our study. Considering either the minimum or the maximum additional profit from biochar use, the NPV is negative and therefore economically unviable. The values ranged from −5,705 to −5,572. We also performed a simulation for financing of a biochar addition using the available fund from Low Carbon Agriculture Plan (Agricultura de Baixo Carbono, in Portuguese, ABC Plan^35^). Considering the biochar production costs at 15t/ha rate and three different scenarios to estimate the minimum value of the carbon price we found that carbon price would need to range from US$ 53.09 to US$ 78.72 to make biochar use economically viable.

## Discussion

Good pasture quality is fundamental for food production and initiatives to improve pasture quality have been promoted by public and private sectors^6,37^. Intensification of cattle ranching is happening in vast areas of Brazilian pasturelands but the question on how this intensification should be performed in the most sustainable way remains^4^. Results from this study show that incorporating biochar into pastureland soil could improve pasture productivity and positively impact a range of indicators for forage and soil quality. It could also contribute to better waste management since *Gliricidia* is one of residue biomass sources in Brazil such as Sabiá (*Mimosa caesalpiniaefolia*) or residues from pruning. Considering farmers’ practice in Brazil who already apply charred residues into soil, and given that burning of organic residues is common throughout rural areas, biochar production could offer an alternative to business-as-usual (see video associated with this paper).

The increase in biomass productivity seen here can be ascribed to the positive changes in soil properties. A significant increase in soil pH in the treatments containing biochar was observed for both pot and field trials, and is consistent with other studies that have been carried out on acidic degraded soils^37,38^. High acidity of degraded pasturelands is one of the principal limitations to increasing cattle ranching productivity. Biochar provides not only a liming effect, but also increases the content of macronutrients such as P and K as was observed for both forages in both the pot and field experiments. However, other macronutrients such as Ca and Mg, although also increased in the soils where biochar was added, decreased in the forage biomass in treatments with biochar.  This could be explained by a dilution effect given the higher volumes of biomass (Supplementary Fig. S6) or a antagonistic effect where high K absorption could have interfered with the capacity of grasses to absorb other cations^39^. Treatments with fertilizer did not provide prolonged fertilization effect with respect to Mg and in Brazil, cattle are universally fed magnesium supplements to avoid bone fractures. In this study the positive interaction between biochar and fertilizer observed for *Panicum* corroborates the results from literature that have shown that to maximize biochar effect on agricultural productivity of selected plants, biochar should be used in combination with fertilizer^40^.

Different results were obtained for the pot and field studies. Although patterns observed in the field for some soil parameters (pH, K and P contents) and biomass nutrient contents were consistent with the results from the pot experiment, the increases in aboveground biomass yield in the pot trial were not observed in the field. The results of the field trials, however, must be interpreted with caution, given that the plots were relatively small (2 × 2 m), with few replicates and less harvests. Although the field experiment was initially planned to last six harvest cycles (as for the pot trial), it had to be ended sooner due to severe drought, fire risk and forage shortage for neighbouring cattle. The field trials enabled surveying practical aspects of biochar, such as time and costs involved in biochar production in different types of stoves and field application; aspects which are paramount for implementation of biochar use in practice and its economic and social viability.

Despite literature stating that biochar may be a low-cost soil amendment with a high adoption potential for local farmers^41^, few studies present comprehensive analyses that support such claims. Here we show that although on-farm methods to produce biochar are simple and do not require sophisticated equipment to be installed and run, when the labour costs are taken into account, biochar application at the rate considered in this study is not competitive when compared with other soil amending alternatives. Even with the significant increase in the pasture productivity following the application of biochar and potential additional profits to the farmers with increased meat production, financial viability to produce and apply biochar for a small to medium size farmland remains a great challenge. This is not uncommon for emerging technologies, but the need for further research and investment should be highlighted and the use of biochar in practice should not be promoted without full transparency and a thorough cost-benefit analysis. Possible alternatives to diminish costs are using commercially produced biochar, applying biochar at lower doses (depending whether productivity increase will follow) or using biochar for a more profitable land use, as it is difficult for a small to medium-holder farmers to be economically viable when cattle ranching for meat production is considered^42^. Given additional benefits from biochar use such as carbon sequestration, farmers could also benefit from Payments for Ecosystem Services to leverage the costs and contribute to global goals to combat climate change. If payments for carbon were used to subsidize biochar use, carbon price would need to range from US$ 53.09 to US$ 78.72. Interestingly, to achieve the goals of the Paris Agreement, the carbon price would have to be in the range of US$ 40 to US$ 80 in 2020 and US$ 50 to US$ 100 in 2030^43^. In Brazil, one of the possibilities for encouraging the use of biochar would be through ‘Low Carbon Agriculture’ funding, since biochar is one of the possibilities for improving soil quality and may contribute to the reduction of greenhouse gases.

Improving land management of Brazilian farmlands is important because local and global ecosystem services should be maintained and deforestation of remaining native ecosystems should be limited^44^. Since smallholder farmers often search for alternative methods to increase productivity and reverse land degradation, biochar could help improving soil properties while increasing the provision of other important ecosystem services. Here, food production and carbon sequestration were valued as they reflect soil provisioning and regulating services, respectively. There are other potential ecosystem services stemming from biochar use which were not valuated here, such as supporting service of nutrient cycling (shown in our experiments through increases in P, K, Ca, Mg, for example) and regulating service through liming effect and increase in soil pH. Another crucial ecosystem service is water provision, and although biochar has been shown to improve water retention^44^ this was not observed in our study. It is important therefore that researchers analyse the extent to which biochar can contribute to a wider range of soil ecosystem services, as to date such analysis remain overlooked. Adequate and comprehensive valuation of biochar ecosystem services is largely excluded from published studies and environmental impact assessments and therefore rarely considered in decision-making and environmental policies. This study has highlighted the importance of considering biochar in the context of ecosystem services to better estimate biochar potential costs and benefits, and its place in wider context of soil contribution to human well-being.

## Supplementary information

Video - Practical Application of Biochar in Brazil

Supplementary information
