# Supplementary material for: Biochar amendment improves degraded pasturelands in Brazil: environmental and cost-benefit analysis
Source: Sci Rep. 2019 Aug 19;9:11993. doi: 10.1038/s41598-019-47647-x (PMC6700309; doi:10.1038/s41598-019-47647-x)
Supplement: Supplementary file 2 — Supplementary information [file 41598_2019_47647_MOESM2_ESM.docx]

**Biochar amendment improves degraded pasturelands in Brazil: environmental and cost-benefit analysis**

# Agnieszka E Latawiec^1,2,3,4,5*^, Bernardo BN Strassburg^1,2,6^, André B Junqueira^1,2^, Ednaldo Araujo^7^, Luiz Fernando de Moraes^7^, Helena AN Pinto^1,2,6^, Ana Castro^1,2^, Marcio Rangel^2^, Gustavo A Malaguti^1,2^, Aline F Rodrigues^1,2^, Luis Gustavo Barioni^9^, Etelvino H Novotny^10^, Gerard Cornelissen^11^, Maiara Mendes^1,2,3^, Nilcileny Batista^8^, Jose Guilherme Guerra^7^, Everaldo Zonta^8^, Catarina Jakovac^2^, Sarah Hale^11^

**Supplementary information**

**
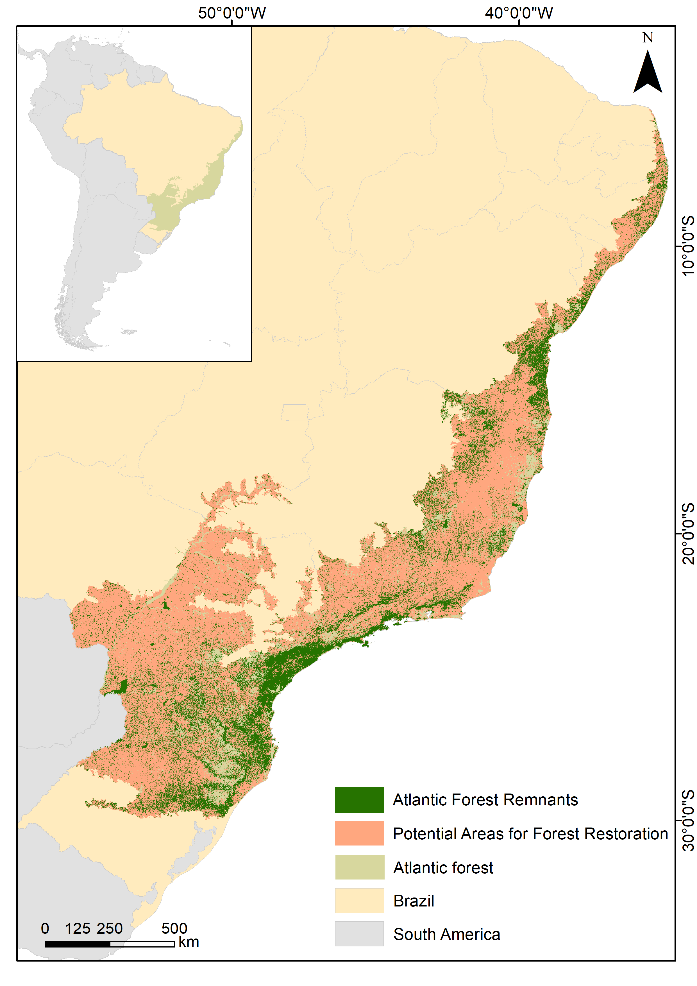
**

**Supplementary Figure S1 |** Map of the Atlantic Forest biome with indication of potential areas for restoration; forest remnants; and original limits of the Atlantic Forest. Source: MapBiomas (2016)¹ and Ministry of the Environment of Brazil (2018).

**Supplementary Video S1 |** Practical applications of biochar in Brazil

**Supplementary Methods |** Climate information of the study sites

According to the Köppen (1980) classification, the climate of the region is of ‘Tropical wet and dry or savanna’ (Aw), with average annual temperature of 24° C and average annual precipitation of 1.483 mm. The variation in temperature and precipitation over the duration of the study are in Supplementary Figure S2. The dry period is between April and September, while the rainy season usually starts in October or November and finishes in March. The average annual insolation is 2.527 hours, the average annual evaporation is 1.576 mL and the average relative air humidity is 69%. Experiments were conducted on soil classified according to FAO (1998)^2^ as Planosol (91.6% sand, 3.4% silt and 5.0% clay), which occupy approximately 130 million hectares globally and are commonly found under tropical pasturelands.

**Supplementary Figure S2 |** Variation in temperature and precipitation over the duration of the study


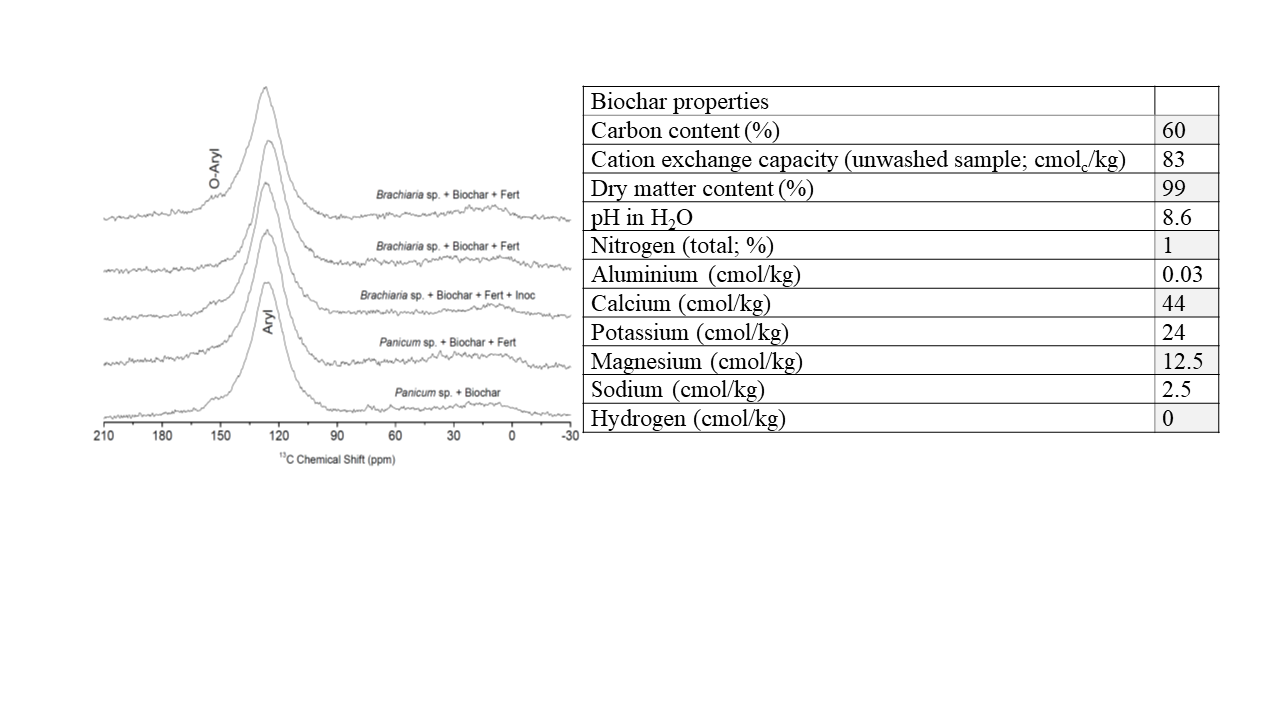


**Supplementary Figure S3** | Presence of recalcitrant carbon in soil samples upon biochar addition (left); Properties of biochar used in this study (right).

**Supplementary methods** **|** Root analysis

Three out of five replicates were randomly selected from each treatment. The root system was washed and stored in water pots in a refrigerator at 10°C. The roots were then evenly spread in a transparent acrylic tray (30 cm x 20 cm) with a water layer of 5 mm and scanned at a resolution of 600 dpi (dots per inch) using an Epson Expression 10000XL scanner with additional light unit. The root images were then converted to an eight-bit grayscale format and analyzed using the software WinRhizo Arabidopsis, which uses the Regent non-statistical method for measurements of root morphology^3,4^. Having the morphological parameters, the roots were dried in a forced air circulation oven at 60°C until they reached constant weight, then weighed and finely ground in a Willey mill (20mesh; 0.5mm). To determine P, K, Ca, Mg, S, Zn, Cu, Mn and Fe, digestion with nitric acid was performed according to USEPA method 3052^5^. P was determined by colorimetry, K by flame emission photometry, Ca, Mg, S, Zn, Cu, Mn and Fe by atomic absorption spectrophotometry and S by turbidimetry. B was determined by dry digestion and quantified using the curcumin method^6^. C and total N were determined using dry combustion and with an elemental LECO Corporation analyzer, model TruSpec CHN.

**Supplementary methods |** Dates of biomass collection in the pot experiment

Beginning of experiment: February 6^th^ 2015

1^st^ harvest : April 15 2015

2^nd^ harvest: June 25 2015

3^rd^ harvest: September 1^st^ 2015

4^th^ harvest: November 2^nd^2015

5^th^ harvest: February 2^nd^ 2016

6^th^ harvest: May 5^th^ 2016

**Supplementary methods |** Soil Sampling and analysis

For pot experiments, soils were analysed at the beginning and at the end of the experiment (November 25, 2014 and May 5, 2016). In the field trial, soil samples were collected at the end of two harvest cycle of each forage (exact dates for both forages are in supplementary methods – dates of biomass collection in the pot experiment). Five subsamples along two diagonal lines through each plot were collected and pooled into one composite sample. Soil samples were homogenized and sieved to 2 mm and soil pH values were measured in water (pH H_2_O), 0.01 M CaCl2 (pH CaCl_2_) and 1 M KCl (pH KCl) at 1:2.5 soil/water or solution ratio^7^. PF curves were calculated in Richards pressure chamber and the Plant-available water was determined by the difference between water retention at 0.10 and 15 atm. Potassium (K), sodium (Na), zinco (Zn), Copper (Cu), Manganese (Mn), Iron (Fe) were extracted by Mehlich^-1^. Phosphorus (P) was extracted both Mehlich^-1^ and resin. And the remaining P was also determined through a solution of CaCl_2_ 10 mmol L^-1^ containing 60 mg L^-1^ of P ^8^. Calcium (Ca) and magnesium (Mg) were extracted with 1.0 mol L^-1^ KCl and determined by atomic absorption spectrophotometry. Based on K^+^, Ca2^+^, Mg2^+^, Al3^+^ and H+Al contents, it were calculated sum of bases (SB), potential (T) and effective (t) cation exchange capacity calculated (CEC), base saturation (V) and aluminium (m) indexes according to Embrapa (1997)^7^. Total nitrogen of the field samples was determined using the Kjedahl method. Organic matter was extracted through a solution of NaCr_2_O_7_ and H_2_SO^4 9^. C, H and N percent in the pot experiment was determined using the Dumas method via dry combustion of 5.0 mg (±0.1 mg) soil in an element analyzer, PerkinElmer 2400. Acetanilide was used as reference material. The analysis and methods applied according to each type of sample are presented are on the table below.

Types of soil analysis and methods applied according to the type of samples. The methodological analyzes were separated according to the sample types. For each type of sample, certain analyzes and methods were used.

| Analysis | Applied method |
| --- | --- |
| Analytical methods: Pot expriments (composite samples) | |
| Soil pH (1:2.5 soil/solution ratio6) | Measurement in water (pH H2O) |
|  | Measurement in 0.01 M CaCl2 (pH CaCl2) |
|  | Measurement in 1 M KCl (pH KCl) |
| Plant-available water | Difference between water retention at 0.10 and 15 atm |
| Potassium (K) | Extraction by Mehlich -1 |
| Sodium (Na) |  |
| Zinc (Zn) |  |
| Copper (Cu) |  |
| Manganese (Mn) |  |
| Iron (Fe) |  |
| Phosphorus (P) | Extraction by Mehlich -1 and resin |
|  | Determined using a sol. Of CaCl2 10 mmol L-1 containing 60 mg L-1 of P7 |
| Calcium (Ca) | Extraction by 1.0 mol L-1 KCl and determination by atomic absorption spectrophotometry |
| Magnesium (Mg) |  |
| Analytical methods: Field samples | |
| Total nitrogen | Kjedahl |
| Organic matter | Extraction using a sol. Of NaCr2O7 and H2SO4^8^ |
| Carbon (C) % | Dumas method via dry combustion of 5.0 (± 0.1 mg) soil in a Perkin Elmer 2400 |
| Hydrogen (H) % |  |
| Nitrogen (N) % |  |
| Analytical methods: Based on K+, Ca2+, Mg2+, Al3+ and H+Al contents | |
| Sum of bases (SB) | Embrapa (1996)^7^ |
| Potential (T) cation exchange capacity |  |
| Effective (t) cation exchange capacity |  |
| Base saturation (V) |  |
| Aluminium (m) index |  |

Five subsamples along two diagonal lines through each plot were collected and pooled into one composite sample. Soil samples were homogenized and sieved to 2 mm and soil pH values were measured in water (pH H_2_O), 0.01 M CaCl2 (pH CaCl_2_) and 1 M KCl (pH KCl) at 1:2.5 soil/water or solution ratio^6^. PF curves were calculated in Richards pressure chamber and the Plant-available water was determined by the difference between water retention at 0.10 and 15 atm. Potassium (K), sodium (Na), zinco (Zn), Copper (Cu), Manganese (Mn), Iron (Fe) were extracted by Mehlich^-1^. Phosphorus (P) was extracted both Mehlich^-1^ and resin. And the remaining P was also determined through a solution of CaCl_2_ 10 mmol L^-1^ containing 60 mg L^-1^ of P ^8^. Calcium (Ca) and magnesium (Mg) were extracted with 1.0 mol L^-1^ KCl and determined by atomic absorption spectrophotometry. Based on K^+^, Ca2^+^, Mg2^+^, Al3^+^ and H+Al contents, it were calculated sum of bases (SB), potential (T) and effective (t) cation exchange capacity calculated (CEC), base saturation (V) and aluminium (m) indexes according to Embrapa (1997)^7^. Total nitrogen of the field samples was determined using the Kjedahl method. Organic matter was extracted through a solution of NaCr_2_O_7_ and H_2_SO^4 9^. C, H and N percent in the pot experiment was determined using the Dumas method via dry combustion of 5.0 mg (±0.1 mg) soil in an element analyzer, PerkinElmer 2400. Acetanilide was used as reference material.

**Supplementary Methods |** Meat profit calculation from biomass

Variation of the biomass generated from the treatment in relation to the control (ton). The variation of the Brachiaria for each treatment: inoculant = -0.000004; fertilizer = 0.000005; fertilizer+inoculant = 0.000010; biochar = 0.000011; biochar+fertilizer = 0.000008; biochar+fertilizer+inoculant = 0.000008.

**Supplementary Table S1 |** Information about the biochar applied to the pots and the carbon derived from it.

|  | **Weight biochar sieve (g)** | **Carbon sieve (g)** | **Biochar applied (g)** | **% Carbon applied (g)** | **% Soil carbon** | **Pot total volume (l)** | **Biochar application volume (l)** |
| --- | --- | --- | --- | --- | --- | --- | --- |
| **Pot 31** | 33 | 21.582 | 75 | 49.05 | 0.98 | 9 | 5 |
| **Pot 32** | 26 | 17.004 | 75 | 49.05 | 0.81 | 9 | 5 |
| **Pot 36** | 28 | 18.312 | 75 | 49.05 | 0.44 | 9 | 5 |
| **Pot 50** | 22 | 14.388 | 75 | 49.05 | 0.38 | 9 | 5 |
| **Pot 55** | 26 | 17.004 | 75 | 49.05 | 0.61 | 9 | 5 |
| **Average** | 27 | - | 0.36 | - | - | - | - |

**Supplementary Table S2 |** Costs and gains and the relation between costs and gains with the production of biochar in different kilns, in $

| **Kiln type** | **maximum kiln yield** | **gains with biochar selling** | **maximum cost with the kiln** | **relation between gains and costs (%)** |
| --- | --- | --- | --- | --- |
| Ground kiln | 44.00 | 665.87 | 5,687.16 | 11.71 |
| Traditional brick kiln | 68.44 | 1,035.79 | 5,983.63 | 17.31 |
| Simple drum kiln | 85.56 | 1,294.74 | 5,776.10 | 22.42 |
| Two-drum kiln | 30.56 | 462.41 | 5,175.91 | 8.04 |

**Supplementary Table S3|** Costs of production in different kilns and data on efficiency

| Summary subtotal – Economic Analysis of biochar production | | | |
| --- | --- | --- | --- |
| **Investments** | **Floor kiln** | Subtotal | % investiment |
|  | Equipment and improvements | $ 872.74 | 61.3 |
|  | Feedstock | $ 1.64 | 0.1 |
|  | Inputs and others | $ 272.56 | 19.1 |
|  | Support | $ - | 0.0 |
|  | Labor costs and labor charges (per month) | $ 277.58 | 19.5 |
|  | TOTAL | **$ 1,424.51** | 100.0 |
|  | **Traditional brick kiln** | Subtotal | % investiment |
|  | Equipment and improvements | $ 902.14 | 61.9 |
|  | Feedstock | $ 1.25 | 0.1 |
|  | Inputs and others | $ 275.98 | 18.9 |
|  | Support | $ - | 0.0 |
|  | Labor costs and labor charges (per month) | $ 277.58 | 19.1 |
|  | TOTAL | **$ 1,456.95** | 100.0 |
|  | **Simple drum kiln** | Subtotal | % investiment |
|  | Equipment and improvements | $ 950.38 | 62.6 |
|  | Feedstock | $ 1.42 | 0.1 |
|  | Inputs and others | $ 275.98 | 18.2 |
|  | Support | $ 12.82 | 0.8 |
|  | Labor costs and labor charges | $ 277.58 | 18.3 |
|  | TOTAL | **$ 1,518.19** | 100.0 |
|  | **Two-drum kiln** | Subtotal | % investiment |
|  | Equipment and improvements | $ 915.70 | 61.5 |
|  | Feedstock | $ 0.27 | 0.0 |
|  | Inputs and others | $ 272.56 | 18.3 |
|  | Support | $ 22.79 | 1.5 |
|  | Labor costs and labor charges | $ 277.58 | 18.6 |
|  | TOTAL | **$ 1,488.90** | 100.0 |

Feedstock is woody material used in ovens. According to field research, it was estimated that the average density of eucalyptus wood is 0.525 m^3^, the market price of this wood for charcoal or wood was R$ 25.00/m^3^, and the following proportions of wood in each oven:

| **Kilns** | **Feedstock (m3)** |
| --- | --- |
| In the ground stove | 0.23 |
| Traditional brick kiln | 0.18 |
| Simple drum stove | 0.20 |
| Two-drum stove | 0.04 |

**Supplementary Table S4 |** Additional costs of stoves

| **Kilns** | **Additional cost of a kiln** |
| --- | --- |
| In the ground stove | $ 20.66 |
| Traditional brick kiln | $ 326.01 |
| Simple drum stove | $ 57.25 |
| Two-drum stove | $ 58.56 |

**Supplementary Methods |** Labour costs and labor charges

Labor costs and labor charges – Monthly cost of salary, calculated from the average minimum wage of the experiment period, $ 271.64, in 2015, and $ 281.65, in 2016, counting too the 13^th^ salary. Thus, the total cost of labor costs and labor charges (0.7% of salary) is the average monthly cost multiplied by months worked plus 13^th^ salary (n = 16,13). So, the total labor costs and labor charges in all experiment period is $ 4,478.24. However, in the case of the use of family labor, in particular, in small properties, this value can be given as zero. So, there are without labor costs and labor charges in production of biochar. As a result, production is less expensive, but is still high within the gains generated from the sale, resulting yields of each kiln or compared to the need for labor and kiln to produce 15t/ha of biochar.

**Supplementary Methods |** Cost-benefit analysis comparing biochar with fertilizer, inoculant and lime

Prices of biofuel (same price of coal) and fertilizer were obtained in field research. In contrast, the prices of limestone and inoculant were measured from IEA / SP data and added the cost of transportation from São Paulo to Seropédica. Biofuel price was R$1.50/kg and fertilizer price was estimated through the utilized proportion of thermophosphate, (562.5 kg) – which price was R$1.07 – and potassium sulphate (120 kg), which price was R$21.10/kg. Transportation cost was considered as “zero”, since the values ​​are of the companies present in the region. At the same time, values were converted to US dollars by using the average exchange rate of the study period, obtaining a value of R$ 3.51/U$. As a result, the total cost of applying biochar was estimated at U$ 6,410.26 and fertilizer at U$ 892.84. Regarding the prices of limestone and inoculant, the data used came from IEA / SP^9^, which has the prices of these inputs from April 2015. An average of the period from April 2015 to May 2016 was calculated, excluding the months of February and March 2015 due to lack of information. Therefore, the average price of limestone was R$ 78.58/t and inoculant was R$ 1.57/g. At the same time, since the data was generated in a survey carried out in São Paulo, it was necessary to account for transportation costs if the producer had the need to purchase the product. For that, the freight estimated by ANTT (2018)^11^ (R$ 0.95/km) was applied, considering the distance of 370km between São Paulo and Seropédica and a truck with capacity of 41.5t, thus obtaining the value per t of the product of about R$ 8.47. The exchange rate was also applied. Therefore, limestone costs were measured at U$ 74.40 and with inoculant, U$ 472.93.

**Supplementary Methods |** Calculation of minimum carbon price

In order to calculate the minimum carbon price to make the use of biochar economically viable through funding from the ABC Plan (10 years of project financing and 5 years of grace period), with an estimated cost of U$ 6,410.26 and a rate of 6%/year, the net present value (NPV) was estimated in three different scenarios for the gain from beef production and considering 91 tCO2_e_ sequestrated to measure the value of the carbon price needed to the NPV equals a zero which is always negative in all scenarios.

$NPV=\sum_{t=1}^{120} \frac{CF}{{(1+i)}^{t}}-I$, where

NPV = net present value;

CF = cash flow;

i = Discount rate, in this case, 6%/year;

t = Number of time periods, 120 months (10 years)​;

I = initial investment

Scenario I (A): the gains with the use of biochar on the production of meat only last for the experiment period (approximately 15 months) and with the minimum productivity of the grass Brachiaria cv. Marandu of 10 tMS/ha.

Scenario I (B): the gains with the use of biochar on the production of meat only last for the experiment period (approximately 15 months) and with the maximum productivity of the grass Brachiaria cv. Marandu of 17 tMS/ha.

Scenario II (A): the gains with the use of biochar on the production of meat are maintained until the end of the project (120 months) and with the minimum productivity of the grass Brachiaria cv. Marandu of 10 tMS/ha.

Scenario II (B): the gains with the use of biochar on the production of meat are maintained until the end of the project (120 months) and with the maximum productivity of the grass Brachiaria cv. Marandu of 17 tMS/ha.

Scenario III (A): the gains with the use of biochar on the production of meat have a linear tendency of loss from the 16th month until zero at the end of the project (120 months) and with the minimum productivity of the grass Brachiaria cv. Marandu of 10 tMS/ha.

Scenario III (B): the gains with the use of biochar on the production of meat have a linear endency of loss from the 16th month until zero at the end of the project (120 months) and with the maximum productivity of the grass Brachiaria cv. Marandu of 17 tMS/ha.

**Supplementary Table S5 |** Different scenarios to estimate the minimum value of the carbon price

| **Scenario** | **NPV** | **Minimum carbono price** |
| --- | --- | --- |
| IA | -7,164.93 | 78.72 |
| IB | -7,024.55 | 77.17 |
| IIA | -5,875.32 | 64.55 |
| IIB | -4,832.22 | 53.09 |
| IIIA | -6,516.43 | 71.59 |
| IIIC | -5,922.10 | 65.06 |

**Supplementary Table S6 |** Results of generalized linear models showing the effects of Biochar, Fertilizer and Inoculant (and their interactions) on the dry and fresh root biomass of *Brachiaria* and *Panicum* forage grasses planted in a greenhouse experiment. Est – estimated coefficient; SE – Standard error of the estimate. Significant p-values are highlighted in bold.

|  |  | **Dry biomass** | | | | **Fresh biomass** | | | |
| --- | --- | --- | --- | --- | --- | --- | --- | --- | --- |
|  |  | **Est.** | **SE** | **t** | **p** | **Est.** | **SE** | **t** | **p** |
| **Forage grass: Brachiaria** | |  |  |  |  |  |  |  |  |
|  | Intercept | 1.049 | 0.519 | 2.02 | 0.061 | 2.665 | 0.465 | 5.74 | 0.000 |
|  | Biochar | 1.009 | 0.735 | 1.37 | 0.189 | 0.917 | 0.657 | 1.40 | 0.182 |
|  | Fertilizer | -0.601 | 0.735 | -0.82 | 0.425 | -0.515 | 0.657 | -0.78 | 0.445 |
|  | Inoculant | -0.246 | 0.735 | -0.34 | 0.742 | -0.248 | 0.657 | -0.38 | 0.711 |
|  | Biochar*Fertilizer | 0.219 | 1.039 | 0.21 | 0.835 | 0.100 | 0.929 | 0.11 | 0.916 |
|  | Biochar*Inoculant | 0.074 | 1.039 | 0.07 | 0.944 | -0.051 | 0.929 | -0.05 | 0.957 |
|  | Fertilizer*Inoculant | 1.043 | 1.039 | 1.00 | 0.330 | 0.952 | 0.929 | 1.02 | 0.321 |
|  | Biochar*Fertilizer*Inoculant | -1.220 | 1.469 | -0.83 | 0.419 | -0.933 | 1.314 | -0.71 | 0.488 |
| **Forage grass: Panicum** | |  |  |  |  |  |  |  |  |
|  | Intercept | 3.589 | 0.287 | 12.51 | 0.000 | 2.222 | 0.267 | 8.31 | 0.000 |
|  | Biochar | 0.575 | 0.406 | 1.42 | 0.194 | 0.403 | 0.378 | 1.07 | 0.318 |
|  | Fertilizer | 0.422 | 0.406 | 1.04 | 0.329 | 0.346 | 0.378 | 0.92 | 0.387 |
|  | Biochar*Fertilizer | -0.079 | 0.574 | -0.14 | 0.894 | 0.105 | 0.535 | 0.20 | 0.849 |
|  |  |  |  |  |  |  |  |  |  |

**Supplementary Table S7 |** Results of mixed effect models with repeated measures on the effects of Biochar, Fertilizer and Inoculant (and their interactions) on the dry and fresh biomass of *Brachiaria* and *Panicum* forage grasses planted in a field experiment. SS – sum of squares; MSS – mean sum of squares; DF – degrees of freedom; R^2^c – conditional R^2^; R^2^m – marginal R^2^.

|  |  | **Dry biomass** | | | | | | **Fresh biomass** | | | | | |
| --- | --- | --- | --- | --- | --- | --- | --- | --- | --- | --- | --- | --- | --- |
| **Factor** | | **SS** | **MSS** | **DF** | **DenDF** | **F** | **p** | **SS** | **MSS** | **DF** | **DenDF** | **F** | **p** |
| **Forage grass: Brachiaria** | |  |  |  |  |  |  |  |  |  |  |  |  |
|  | Biochar | 0.17 | 0.17 | 1 | 32.0 | 0.91 | 0.348 | 0.05 | 0.05 | 1 | 32.0 | 0.24 | 0.629 |
|  | Fertilizer | 0.08 | 0.08 | 1 | 32.0 | 0.41 | 0.524 | 0.13 | 0.13 | 1 | 32.0 | 0.67 | 0.421 |
|  | Inoculant | 0.02 | 0.02 | 1 | 32.0 | 0.09 | 0.767 | 0.03 | 0.03 | 1 | 32.0 | 0.17 | 0.683 |
|  | Time | 20.61 | 20.61 | 1 | 32.0 | 112.40 | **0.000** | 14.64 | 14.64 | 1 | 32.0 | 72.66 | **0.000** |
|  | Biochar*Fertilizer | 0.01 | 0.01 | 1 | 32.0 | 0.04 | 0.838 | 0.02 | 0.02 | 1 | 32.0 | 0.08 | 0.774 |
|  | Biochar*Inoculant | 0.08 | 0.08 | 1 | 32.0 | 0.45 | 0.509 | 0.12 | 0.12 | 1 | 32.0 | 0.58 | 0.453 |
|  | Fertilizer*Inoculant | 0.00 | 0.00 | 1 | 32.0 | 0.01 | 0.934 | 0.02 | 0.02 | 1 | 32.0 | 0.08 | 0.778 |
|  | Biochar*Time | 0.25 | 0.25 | 1 | 32.0 | 1.34 | 0.255 | 0.37 | 0.37 | 1 | 32.0 | 1.85 | 0.184 |
|  | Fertilizer*Time | 0.00 | 0.00 | 1 | 32.0 | 0.00 | 0.993 | 0.00 | 0.00 | 1 | 32.0 | 0.01 | 0.926 |
|  | Inoculant*Time | 0.11 | 0.11 | 1 | 32.0 | 0.62 | 0.438 | 0.14 | 0.14 | 1 | 32.0 | 0.69 | 0.413 |
|  | Biochar*Fertilizer*Inoculant | 0.00 | 0.00 | 1 | 32.0 | 0.00 | 0.989 | 0.00 | 0.00 | 1 | 32.0 | 0.00 | 0.972 |
|  | Biochar*Fertilizer*Time | 0.00 | 0.00 | 1 | 32.0 | 0.02 | 0.887 | 0.00 | 0.00 | 1 | 32.0 | 0.02 | 0.881 |
|  | Biochar*Inoculant*Time | 0.00 | 0.00 | 1 | 32.0 | 0.01 | 0.934 | 0.00 | 0.00 | 1 | 32.0 | 0.01 | 0.944 |
|  | Fertilizer*Inoculant*Time | 0.11 | 0.11 | 1 | 32.0 | 0.60 | 0.444 | 0.16 | 0.16 | 1 | 32.0 | 0.80 | 0.379 |
|  | Biochar*Fertilizer*Inoculant*Time | 0.04 | 0.04 | 1 | 32.0 | 0.23 | 0.635 | 0.02 | 0.02 | 1 | 32.0 | 0.09 | 0.770 |
|  | R^2^m | 0.71 |  |  |  |  |  | 0.62 |  |  |  |  |  |
|  | R^2^c | 0.71 |  |  |  |  |  | 0.62 |  |  |  |  |  |
| **Forage grass: Panicum** | |  |  |  |  |  |  |  |  |  |  |  |  |
|  | Biochar | 0.00 | 0.00 | 1 | 12.0 | 0.02 | 0.891 | 0.00 | 0.00 | 1 | 12.0 | 0.03 | 0.873 |
|  | Fertilizer | 0.00 | 0.00 | 1 | 12.0 | 0.01 | 0.921 | 0.00 | 0.00 | 1 | 12.0 | 0.04 | 0.850 |
|  | Time | 21.48 | 10.74 | 2 | 24.0 | 279.27 | **<2e-16** | 23.00 | 11.50 | 2 | 24.0 | 428.54 | **<2e-16** |
|  | Biochar*Fertilizer | 0.01 | 0.01 | 1 | 12.0 | 0.17 | 0.687 | 0.00 | 0.00 | 1 | 12.0 | 0.08 | 0.783 |
|  | Biochar*Time | 0.02 | 0.01 | 2 | 24.0 | 0.21 | 0.812 | 0.03 | 0.01 | 2 | 24.0 | 0.52 | 0.599 |
|  | Fertilizer*Time | 0.02 | 0.01 | 2 | 24.0 | 0.20 | 0.820 | 0.04 | 0.02 | 2 | 24.0 | 0.81 | 0.458 |
|  | Biochar*Fertilizer*Time | 0.02 | 0.01 | 2 | 24.0 | 0.25 | 0.779 | 0.00 | 0.00 | 2 | 24.0 | 0.03 | 0.969 |
|  | R^2^m | 0.84 |  |  |  |  |  | 0.85 |  |  |  |  |  |
|  | R^2^c | 0.93 |  |  |  |  |  | 0.95 |  |  |  |  |  |

**Supplementary Table** **S8 |** Loadings of the variables included in a Principal Components Analysis based on leaf nutrient parameters measured in a greenhouse experiment with two forage grasses (*Panicum* and *Brachiaria*). Larger values indicate variables that are most strongly correlated with the principal components. Loadings with values larger than 0.6 are highlighted in bold.

| **Variable** | **PC1** | **PC2** | **PC3** |
| --- | --- | --- | --- |
| N | **0.81** | 0.14 | -0.14 |
| P | **0.73** | 0.51 | 0.14 |
| K | -0.05 | **0.86** | 0.04 |
| Ca | 0.52 | **-0.61** | 0.27 |
| Mg | **0.79** | -0.28 | -0.12 |
| S | 0.51 | 0.48 | -0.39 |
| Cu | 0.54 | -0.15 | -0.33 |
| Fe | 0.48 | -0.36 | 0.43 |
| Mn | -0.02 | **-0.75** | 0.02 |
| Zn | -0.19 | -0.51 | **-0.68** |

**Supplementary Table** **S9 |** Results of the mixed effect models with six repeated measures on the effects of Biochar, Fertilizer and Inoculant (and their interactions) on the leaf nutrient content of *Brachiaria* and *Panicum* planted in a pot experiment. PC1 and PC2 are the first two axes of a Principal Components Analysis summarizing the variation in leaf nutrient content, explaining 29.1% and 26.8% of the original variation in leaf nutrient parameters, respectively. SS – sum of squares; MSS – mean sum of squares; DF – degrees of freedom; R^2^c – conditional R^2^; R^2^m – marginal R^2^.

|  |  | **Axis PC1** | | | | | | **Axis PC2** | | | | | | |
| --- | --- | --- | --- | --- | --- | --- | --- | --- | --- | --- | --- | --- | --- | --- |
|  | **Treatment** | **SS** | **MSS** | **DF** | **DenDF** | **F** | **p** | | **SS** | **MSS** | **DF** | **DenDF** | **F** | **p** |
| *Forage grass: Brachiaria* | |  |  |  |  |  |  | |  |  |  |  |  |  |
|  | Biochar | 22.83 | 22.83 | 1 | 32.42 | 49.23 | **0.000** | | 93.19 | 93.19 | 1 | 32.28 | 327.92 | **< 2.2e-16** |
|  | Fertilizer | 0.66 | 0.66 | 1 | 32.42 | 1.43 | 0.241 | | 4.81 | 4.81 | 1 | 32.28 | 16.94 | **0.000** |
|  | Inoculant | 0.00 | 0.00 | 1 | 32.42 | 0.01 | 0.924 | | 0.01 | 0.01 | 1 | 32.28 | 0.03 | 0.871 |
|  | Time | 510.80 | 102.16 | 5 | 157.77 | 220.25 | **< 2.2e-16** | | 235.45 | 47.09 | 5 | 157.57 | 165.70 | **< 2.2e-16** |
|  | Biochar*Fertilizer | 0.43 | 0.43 | 1 | 32.42 | 0.93 | 0.343 | | 0.00 | 0.00 | 1 | 32.28 | 0.00 | 0.965 |
|  | Biochar*Inoculant | 0.00 | 0.00 | 1 | 32.42 | 0.01 | 0.923 | | 0.04 | 0.04 | 1 | 32.28 | 0.14 | 0.713 |
|  | Fertilizer*Inoculant | 0.79 | 0.79 | 1 | 32.42 | 1.69 | 0.202 | | 0.02 | 0.02 | 1 | 32.28 | 0.06 | 0.805 |
|  | Biochar*Time | 20.25 | 4.05 | 5 | 157.77 | 8.73 | **0.000** | | 19.22 | 3.84 | 5 | 157.57 | 13.53 | **0.000** |
|  | Fertilizer*Time | 2.32 | 0.46 | 5 | 157.77 | 1.00 | 0.419 | | 9.11 | 1.82 | 5 | 157.57 | 6.41 | **0.000** |
|  | Inoculant*Time | 3.25 | 0.65 | 5 | 157.77 | 1.40 | 0.227 | | 3.08 | 0.62 | 5 | 157.57 | 2.17 | 0.060 |
|  | Biochar*Fertilizer*Inoculant | 0.92 | 0.92 | 1 | 32.42 | 1.98 | 0.169 | | 0.00 | 0.00 | 1 | 32.28 | 0.01 | 0.931 |
|  | Biochar*Fertilizer*Time | 4.04 | 0.81 | 5 | 157.77 | 1.74 | 0.128 | | 10.04 | 2.01 | 5 | 157.57 | 7.06 | **0.000** |
|  | Biochar*Inoculant*Time | 1.98 | 0.40 | 5 | 157.77 | 0.85 | 0.513 | | 3.47 | 0.69 | 5 | 157.57 | 2.44 | **0.037** |
|  | Fertilizer*Inoculant*Time | 2.79 | 0.56 | 5 | 157.77 | 1.20 | 0.311 | | 0.60 | 0.12 | 5 | 157.57 | 0.42 | 0.833 |
|  | Biochar*Fertilizer*Inoculant*Time | 2.99 | 0.60 | 5 | 157.77 | 1.29 | 0.272 | | 0.40 | 0.08 | 5 | 157.57 | 0.28 | 0.924 |
|  | R^2^m | 0.82 |  |  |  |  |  | | 0.87 |  |  |  |  |  |
|  | R^2^c | 0.86 |  |  |  |  |  | | 0.90 |  |  |  |  |  |
| *Forage grass: Panicum* | |  |  |  |  |  |  | |  |  |  |  |  |  |
|  | Biochar | 16.84 | 16.84 | 1 | 16.28 | 70.43 | **0.000** | | 70.90 | 70.90 | 1 | 16.42 | 357.09 | **0.000** |
|  | Fertilizer | 0.00 | 0.00 | 1 | 16.28 | 0.00 | 0.997 | | 0.59 | 0.59 | 1 | 16.42 | 2.96 | 0.104 |
|  | Time | 199.93 | 39.99 | 5 | 78.82 | 167.25 | **< 2.2e-16** | | 122.96 | 24.59 | 5 | 78.87 | 123.87 | **< 2.2e-16** |
|  | Biochar*Fertilizer | 0.25 | 0.25 | 1 | 16.28 | 1.06 | 0.317 | | 0.01 | 0.01 | 1 | 16.42 | 0.04 | 0.838 |
|  | Biochar*Time | 6.54 | 1.31 | 5 | 78.82 | 5.47 | **0.000** | | 12.07 | 2.41 | 5 | 78.87 | 12.15 | **0.000** |
|  | Fertilizer*Time | 1.51 | 0.30 | 5 | 78.82 | 1.27 | 0.287 | | 3.33 | 0.67 | 5 | 78.87 | 3.35 | **0.009** |
|  | Biochar*Fertilizer*Time | 3.80 | 0.76 | 5 | 78.822 | 3.18 | **0.011** | | 2.48 | 0.50 | 5 | 78.87 | 2.49 | **0.038** |
|  | R^2^m | 0.89 |  |  |  |  |  | | 0.90 |  |  |  |  |  |
|  | R^2^c | 0.89 |  |  |  |  |  | | 0.91 |  |  |  |  |  |
|  |  |  |  |  |  |  |  | |  |  |  |  |  |  |

**Supplementary Table** **S10 |** Results of generalized linear models showing the effects of biochar, fertilizer and inoculant (and their interactions) on root nutrient content measured in greenhouse experiments with two forage grasses (*Panicum* and *Brachiaria*). PC1 and PC2 are the first two axes of a Principal Components Analysis summarizing the variation in root nutrient content, explaining 34.1% e 28.3% of the original variation in root nutrient parameters, respectively. Est – estimated coefficient; SE – Standard error of the estimate. Significant p-values are highlighted in bold.

|  |  | **Axis PC1** | | | | **Axis PC2** | | | |
| --- | --- | --- | --- | --- | --- | --- | --- | --- | --- |
|  | **Treatment** | **Est.** | **SE** | **t** | **p** | **Est.** | **SE** | **T** | **p** |
| *Forage grass: Brachiaria* | |  |  |  |  |  |  |  |  |
|  | Intercept | -0.155 | 0.957 | -0.16 | 0.874 | -0.990 | 1.352 | -0.73 | 0.479 |
|  | Biochar | -1.423 | 1.236 | -1.15 | 0.274 | 1.200 | 1.745 | 0.69 | 0.506 |
|  | Fertilizer | 2.641 | 1.353 | 1.95 | 0.077 | -0.537 | 1.911 | -0.28 | 0.784 |
|  | Inoculant | 1.547 | 1.658 | 0.93 | 0.371 | 1.302 | 2.341 | 0.56 | 0.589 |
|  | Biochar*Fertilizer | -2.899 | 1.747 | -1.66 | 0.125 | -1.159 | 2.468 | -0.47 | 0.648 |
|  | Biochar*Inoculant | -0.254 | 1.992 | -0.13 | 0.901 | -1.628 | 2.814 | -0.58 | 0.574 |
|  | Fertilizer*Inoculant | -2.288 | 2.140 | -1.07 | 0.308 | -0.364 | 3.022 | -0.12 | 0.906 |
|  | Biochar*Fertilizer*Inoculant | 1.804 | 2.650 | 0.68 | 0.510 | 0.058 | 3.742 | 0.02 | 0.988 |
| *Forage grass: Panicum* | |  |  |  |  |  |  |  |  |
|  | Intercept | 0.840 | 0.964 | 0.87 | 0.409 | 0.736 | 0.638 | 1.15 | 0.282 |
|  | Biochar | -1.339 | 1.363 | -0.98 | 0.355 | 0.661 | 0.902 | 0.73 | 0.485 |
|  | Fertilizer | 2.143 | 1.363 | 1.57 | 0.155 | 1.088 | 0.902 | 1.21 | 0.262 |
|  | Biochar*Fertilizer | -3.419 | 1.928 | -1.77 | 0.114 | -0.964 | 1.275 | -0.76 | 0.471 |
|  |  |  |  |  |  |  |  |  |  |

**Supplementary Table S11 |** Loadings of the variables included in a Principal Components Analysis based on root nutrient parameters measured in a greenhouse experiment with two forage grasses (*Panicum* and *Brachiaria*). Larger values indicate variables that are most strongly correlated with the principal components. Loadings with values larger than 0.6 are highlighted in bold.

| **Variable** | **PC1** | **PC2** | **PC3** |
| --- | --- | --- | --- |
| N | -0.29 | **-0.65** | 0.29 |
| P | **-0.85** | 0.03 | -0.27 |
| K | **-0.74** | 0.00 | 0.42 |
| Ca | **-0.78** | -0.05 | 0.23 |
| Mg | **-0.91** | -0.18 | 0.14 |
| S | -0.53 | **-0.68** | -0.24 |
| Cu | -0.35 | 0.31 | -0.70 |
| Fe | **-0.61** | **0.66** | -0.15 |
| Zn | -0.21 | -0.43 | -0.38 |
| Mn | **-0.68** | 0.52 | 0.06 |
| B | -0.17 | **-0.83** | 0.02 |
| C | 0.10 | **-0.89** | -0.24 |
|  |  |  |  |


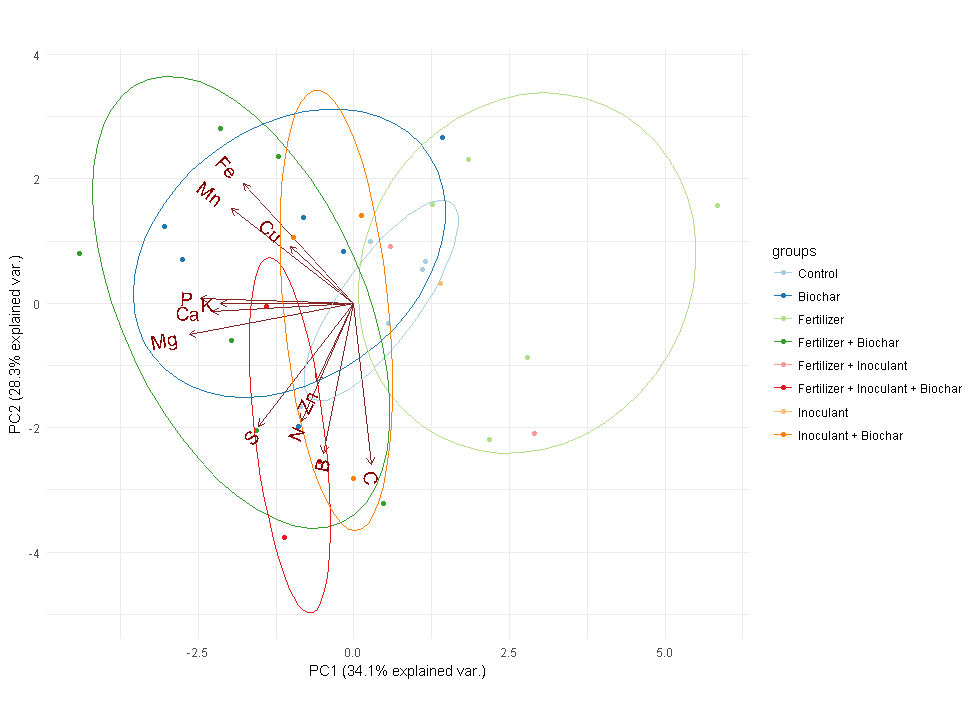
**Supplementary Figure S4 |** Principal Components Analysis of root nutrient content measured in a greenhouse experiment with two forage grasses (*Panicum* and *Brachiaria*). Each point in the figure represents a sample (vase), and their colors indicate the different treatments applied to the vases. Vectors indicate the soil variables included in the PCA, and their length and direction indicate the magnitude and direction in which they contribute to the ordination, respectively. Values between brackets indicate the percentage of the variation in the original dataset that is explained by axes PC1 and PC2.

**Supplementary Table S12 |** Results of the mixed effect models with repeated measures on the effects of Biochar, Fertilizer and Inoculant (and their interactions) on the leaf nutrient content of *Brachiaria* and *Panicum* planted in a field experiment. PC1 and PC2 are the first two axes of a Principal Components Analysis summarizing the variation in leaf nutrient content, explaining 45.7 and 19.6% of the original variation in leaf nutrient parameters, respectively. SS – sum of squares; MSS – mean sum of squares; DF – degrees of freedom; R^2^c – conditional R^2^; R^2^m – marginal R^2^.

|  |  | **Axis PCA1** | | | | | | **Axis PCA2** | | | | | |
| --- | --- | --- | --- | --- | --- | --- | --- | --- | --- | --- | --- | --- | --- |
|  | **Treatment** | **SS** | **MSS** | **DF** | **DenDF** | **F** | **p** | **SS** | **MSS** | **DF** | **DenDF** | **F** | **p** |
| *Forage grass: Brachiaria* | |  |  |  |  |  |  |  |  |  |  |  |  |
|  | Biochar | 8.25 | 8.25 | 1 | 16.00 | 34.23 | **0.000** | 6.37 | 6.37 | 1 | 16.00 | 20.06 | **0.000** |
|  | Fertilizer | 0.85 | 0.85 | 1 | 16.00 | 3.52 | 0.079 | 0.91 | 0.91 | 1 | 16.00 | 2.87 | 0.110 |
|  | Inoculant | 0.01 | 0.01 | 1 | 16.00 | 0.02 | 0.889 | 0.08 | 0.08 | 1 | 16.00 | 0.26 | 0.616 |
|  | Time | 55.46 | 55.46 | 1 | 16.00 | 230.23 | **0.000** | 110.34 | 110.34 | 1 | 16.00 | 347.74 | **0.000** |
|  | Biochar*Fertilizer | 0.40 | 0.40 | 1 | 16.00 | 1.68 | 0.214 | 0.11 | 0.11 | 1 | 16.00 | 0.35 | 0.561 |
|  | Biochar*Inoculant | 0.03 | 0.03 | 1 | 16.00 | 0.12 | 0.737 | 0.20 | 0.20 | 1 | 16.00 | 0.63 | 0.441 |
|  | Fertilizer*Inoculant | 0.00 | 0.00 | 1 | 16.00 | 0.01 | 0.945 | 0.03 | 0.03 | 1 | 16.00 | 0.09 | 0.770 |
|  | Biochar*Time | 0.34 | 0.34 | 1 | 16.00 | 1.43 | 0.249 | 0.03 | 0.03 | 1 | 16.00 | 0.10 | 0.750 |
|  | Fertilizer*Time | 0.02 | 0.02 | 1 | 16.00 | 0.07 | 0.798 | 0.37 | 0.37 | 1 | 16.00 | 1.15 | 0.299 |
|  | Inoculant*Time | 0.06 | 0.06 | 1 | 16.00 | 0.25 | 0.623 | 0.37 | 0.37 | 1 | 16.00 | 1.16 | 0.297 |
|  | Biochar*Fertilizer*Inoculant | 0.01 | 0.01 | 1 | 16.00 | 0.04 | 0.849 | 0.01 | 0.01 | 1 | 16.00 | 0.04 | 0.840 |
|  | Biochar*Fertilizer*Time | 0.00 | 0.00 | 1 | 16.00 | 0.00 | 0.966 | 0.05 | 0.05 | 1 | 16.00 | 0.15 | 0.700 |
|  | Biochar*Inoculant*Time | 0.10 | 0.10 | 1 | 16.00 | 0.42 | 0.528 | 0.43 | 0.43 | 1 | 16.00 | 1.37 | 0.259 |
|  | Fertilizer*Inoculant*Time | 0.09 | 0.09 | 1 | 16.00 | 0.35 | 0.562 | 0.37 | 0.37 | 1 | 16.00 | 1.17 | 0.296 |
|  | Biochar*Fertilizer*Inoculant*Time | 0.13 | 0.13 | 1 | 16.00 | 0.56 | 0.466 | 0.03 | 0.03 | 1 | 16.00 | 0.11 | 0.748 |
|  | R^2^m | 0.79 |  |  |  |  |  | 0.85 |  |  |  |  |  |
|  | R^2^c | 0.89 |  |  |  |  |  | 0.90 |  |  |  |  |  |
| *Forage grass: Panicum* | |  |  |  |  |  |  |  |  |  |  |  |  |
|  | Biochar | 0.23 | 0.23 | 1 | 12.00 | 0.50 | 0.494 | 0.05 | 0.05 | 1 | 12.00 | 0.19 | 0.671 |
|  | Fertilizer | 0.03 | 0.03 | 1 | 12.00 | 0.07 | 0.797 | 0.00 | 0.00 | 1 | 12.00 | 0.00 | 0.968 |
|  | Time | 149.77 | 74.88 | 2 | 24.00 | 159.40 | **0.000** | 4.20 | 2.10 | 2 | 24.00 | 7.52 | **0.003** |
|  | Biochar*Fertilizer | 0.44 | 0.44 | 1 | 12.00 | 0.94 | 0.351 | 0.28 | 0.28 | 1 | 12.00 | 1.01 | 0.334 |
|  | Biochar*Time | 0.18 | 0.09 | 2 | 24.00 | 0.19 | 0.828 | 0.66 | 0.33 | 2 | 24.00 | 1.18 | 0.326 |
|  | Fertilizer*Time | 0.15 | 0.07 | 2 | 24 | 0.15 | 0.857 | 0.32 | 0.16 | 2 | 24.00 | 0.58 | 0.569 |
|  | Biochar*Fertilizer*Time | 1.014 | 0.51 | 2 | 24 | 1.08 | 0.356 | 0.45 | 0.23 | 2 | 24.00 | 0.81 | 0.458 |
|  | R^2^m | 0.81 |  |  |  |  |  | 0.22 |  |  |  |  |  |
|  | R^2^c | 0.88 |  |  |  |  |  | 0.57 |  |  |  |  |  |
|  |  |  |  |  |  |  |  |  |  |  |  |  |  |

**Supplementary Table S13 |** Loadings of the variables included in a Principal Components Analysis based on leaf nutrient parameters measured in a field experiment with two forage grasses (*Panicum* and *Brachiaria*). Larger values indicate variables that are most strongly correlated with the principal components. Loadings with values larger than 0.6 are highlighted in bold.

| **Variable** | **PC1** | **PC2** | **PC3** |
| --- | --- | --- | --- |
| N | **0.74** | -0.36 | -0.09 |
| P | **0.64** | 0.14 | 0.20 |
| K | **-0.74** | -0.36 | -0.21 |
| Ca | **0.77** | 0.36 | -0.27 |
| Mg | **0.74** | 0.48 | 0.02 |
| S | -0.50 | **0.73** | 0.07 |
| Cu | **0.60** | -0.56 | 0.37 |
| Fe | **0.69** | 0.19 | 0.51 |
| Mn | **0.65** | 0.31 | -0.55 |
| Zn | **-0.63** | 0.57 | 0.33 |
|  |  |  |  |


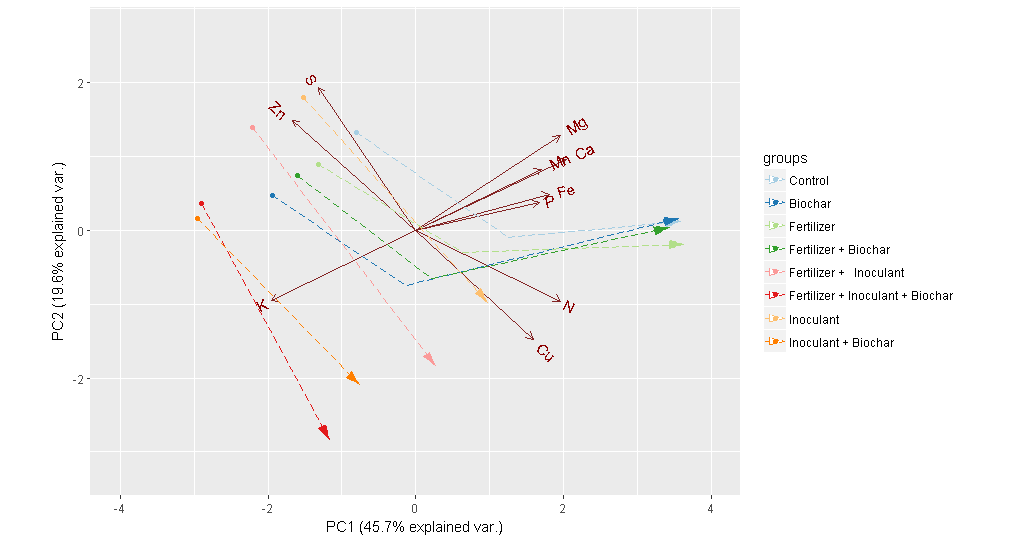


**Supplementary Figure S5 |** Principal Components Analysis of biomass (leaf) nutrient concentration, measured in a field experiment with two forage grasses (*Brachiaria* and *Panicum*). Leaf nutrient concentration was measured in two different times (harvests) for *Brachiaria,* and three different times for *Panicum*. Each point in the figure represents the centroid of five replicates (plots) measured at a given time, their colors indicate the different treatments and the dashed lines connecting the centroids indicate the changes in nutrient contents through time (harvests), with arrows indicating nutrient contents measured at the last harvest. Vectors indicate the soil variables included in the PCA, and their length and direction indicate the magnitude and direction in which they contribute to the ordination, respectively. Values between brackets indicate the percentage of the variation in the original dataset that is explained by axes PC1 and PC2.

**Supplementary Table S14 |** Loadings of the variables included in a Principal Components Analysis based on soil chemical parameters measured in a greenhouse experiment with two forage grasses (*Panicum* and *Brachiaria*). Larger values indicate variables that are most strongly correlated with the principal components. Loadings with values larger than 0.6 are highlighted in bold.

| **Variable** | **PC1** | **PC2** | **PC3** |
| --- | --- | --- | --- |
| N | 0.06 | 0.21 | -0.35 |
| pHCaCl2 | **-0.89** | -0.13 | -0.20 |
| pHKCl | **-0.89** | -0.14 | -0.26 |
| pH | **-0.85** | -0.25 | -0.16 |
| K | **-0.85** | -0.07 | -0.02 |
| P | **-0.60** | 0.40 | -0.08 |
| Na | **-0.61** | 0.38 | 0.02 |
| Ca | **-0.93** | 0.12 | -0.02 |
| Mg | **-0.91** | -0.12 | 0.11 |
| Al | **0.71** | 0.52 | -0.12 |
| Hal | **0.81** | 0.11 | 0.24 |
| Base sums | **-0.98** | 0.03 | 0.00 |
| t | **-0.96** | 0.12 | -0.02 |
| T | **-0.89** | 0.08 | 0.13 |
| V | **-0.97** | -0.03 | -0.11 |
| m | **0.71** | 0.51 | -0.13 |
| MO | -0.26 | 0.33 | 0.43 |
| Pr | -0.13 | 0.48 | 0.19 |
| Zn | -0.45 | **0.62** | -0.29 |
| Fe | **0.72** | 0.19 | -0.41 |
| Mn | -0.46 | 0.41 | -0.40 |
| Cu | 0.20 | -0.26 | -0.19 |
| P2 | **-0.85** | 0.23 | -0.06 |
| total_c | -0.51 | 0.25 | 0.52 |
| total_h | -0.31 | 0.04 | **0.71** |
| total_n | 0.38 | 0.45 | 0.19 |
|  |  |  |  |


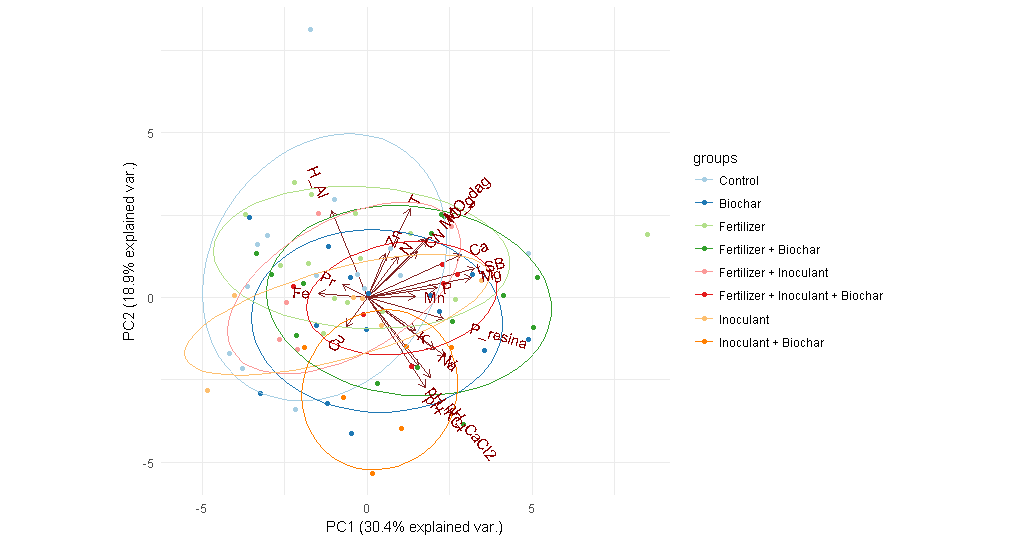


**Supplementary Figure S6** **|** Principal Components Analysis of soil chemical parameters measured in a field experiment with two forage grasses (*Panicum* and *Brachiaria*). Each point in the figure represents a replicate (plot), and their colors indicate the different treatments. Vectors indicate the soil variables included in the PCA, and their length and direction indicate the magnitude and direction in which they contribute to the ordination, respectively. Values between brackets indicate the percentage of the variation in the original dataset that is explained by axes PC1 and PC2.

**Supplementary Table S15 |** Loadings of the variables included in a Principal Components Analysis based on soil chemical parameters measured in a field experiment with two forage grasses (*Panicum* and *Brachiaria*). Larger values indicate variables that are most strongly correlated with the principal components. Loadings with values larger than 0.6 are highlighted in bold.

| **Variable** | **PC1** | **PC2** | **PC3** |
| --- | --- | --- | --- |
| pH_KCl | 0.43 | -0.59 | -0.37 |
| pH_CaCl2 | 0.54 | **-0.68** | -0.20 |
| pH | 0.50 | **-0.77** | 0.09 |
| K | 0.41 | -0.28 | 0.09 |
| P | 0.59 | 0.08 | -0.44 |
| Na | 0.57 | -0.44 | 0.37 |
| Ca | **0.80** | 0.36 | -0.30 |
| Mg | **0.89** | 0.17 | 0.06 |
| H_Al | -0.31 | 0.74 | -0.36 |
| SB | **0.92** | 0.24 | -0.16 |
| t | **0.92** | 0.24 | -0.16 |
| T | 0.37 | **0.75** | -0.39 |
| V | 0.67 | -0.50 | 0.26 |
| MO_dag | 0.50 | 0.50 | **0.63** |
| Pr | -0.21 | 0.11 | -0.38 |
| Zn | 0.16 | 0.37 | -0.52 |
| Fe | -0.41 | 0.04 | 0.26 |
| Mn | 0.41 | 0.01 | -0.43 |
| Cu | -0.18 | -0.24 | -0.19 |
| N | 0.27 | 0.35 | 0.36 |
| P_resina | **0.65** | -0.18 | -0.20 |
| MO_g | 0.51 | 0.49 | **0.63** |
| CN | 0.43 | 0.39 | 0.50 |
|  |  |  |  |

**Supplementary Table S16 |** Results of generalized linear models showing the effects of biochar, fertilizer and inoculant (and their interactions) on soil chemical parameters measured in a field experiment with two forage grasses (*Panicum* and *Brachiaria*). PCA1 and PCA2 are the first two axes of a Principal Components Analysis summarizing the variation in soil parameters, explaining 30.4 and 18.9 % of the original variation in soil parameters, respectively. Est – estimated coefficient; SE – Standard error of the estimate. Significant p-values are highlighted in bold.

|  |  | **Axis PCA1** | | | | **Axis PCA2** | | | |  |
| --- | --- | --- | --- | --- | --- | --- | --- | --- | --- | --- |
|  | **Treatment** | **Est.** | **SE** | **t** | **p** | **Est.** | **SE** | **t** | **p** | |
| *Forage grass: Brachiaria* | |  |  |  |  |  |  |  |  | |
|  | Intercept | -2.196 | 0.972 | -2.26 | 0.029 | -0.857 | 0.663 | -1.29 | 0.204 | |
|  | Biochar | 3.103 | 1.374 | 2.26 | **0.029** | -1.331 | 0.938 | -1.42 | 0.164 | |
|  | Fertilizer | 1.890 | 1.374 | 1.38 | 0.177 | 1.865 | 0.938 | 1.99 | 0.054 | |
|  | Inoculant | 1.270 | 1.374 | 0.93 | 0.361 | 0.333 | 0.938 | 0.36 | 0.725 | |
|  | Biochar*Fertilizer | -2.099 | 1.943 | -1.08 | 0.286 | -0.458 | 1.326 | -0.35 | 0.732 | |
|  | Biochar*Inoculant | -1.796 | 1.943 | -0.92 | 0.361 | -0.953 | 1.326 | -0.72 | 0.477 | |
|  | Fertilizer*Inoculant | -1.663 | 1.943 | -0.86 | 0.397 | -1.050 | 1.326 | -0.79 | 0.433 | |
|  | Biochar*Fertilizer*Inoculant | 2.537 | 2.748 | 0.92 | 0.361 | 2.424 | 1.876 | 1.29 | 0.204 | |
| *Forage grass: Panicum* | |  |  |  |  |  |  |  |  | |
|  | Intercept | -0.626 | 1.049 | -0.60 | 0.555 | 2.244 | 0.624 | 3.60 | **0.001** | |
|  | Biochar | 0.453 | 1.483 | 0.31 | 0.762 | -1.852 | 0.882 | -2.10 | **0.045** | |
|  | Fertilizer | 0.544 | 1.483 | 0.37 | 0.717 | -0.879 | 0.882 | -1.00 | 0.328 | |
|  | Biochar*Fertilizer | 1.329 | 2.098 | 0.63 | 0.532 | 0.897 | 1.248 | 0.72 | 0.478 | |
|  |  |  |  |  |  |  |  |  |  | |

**Supplementary Table S17 |** Estimates of meat production (t/ha) and of the average yields generated for the different for different Brachiaria treatments, during the experiment period (in increasing profit order).

| **Treatment** | **Meat production (t/ha)** | | **Additional profit U$ relative to control per hectare** | |
| --- | --- | --- | --- | --- |
|  | **min** | **max** | **min** | **max** |
| inoculant | 0.24 | 0.40 | -65.97 | -112.16 |
| fertilizer | 0.29 | 0.49 | 82.61 | 140.44 |
| biochar/inoculant | 0.30 | 0.51 | 100.70 | 171.19 |
| biochar/fertilizer/inoculant | 0.31 | 0.53 | 138.88 | 236.10 |
| biochar/fertilizer | 0.31 | 0.53 | 139.45 | 237.06 |
| fertilizer/inoculant | 0.32 | 0.55 | 173.79 | 295.45 |
| biochar | 0.33 | 0.56 | 191.08 | 324.84 |

**References:**

1. MapBiomas Project – 2nd Collection of the Annual Series of Brazilian Land Use and Land Cover Maps, accessed on 23/08/2018 through the link: <http://mapbiomas.org/map#coverage>
2. FAO. World Reference Base for Soil Resources (1998).
3. Arsenault, J.L., Poulcur, S., Messier, C., Guay, R. WinRHlZO™, a root-measuring system with a unique overlap correction method. HortScience, 30(4), 906-906 (1995).
4. Regent instruments. WinRHIZO arabidopsis manual. Régent Instruments Inc. (2012).
5. USEPA. Microwave assisted acid digestion of sediments, sludges, soils and oils. (Technical Resource Document, EPA SW-846/3051A) https://www.epa.gov/sites/production/files/2015-12/documents/3051a.pdf (2015).
6. Malavolta, E., Vitti, G.C., Oliveira, S.A. Avaliação do estado nutricional das plantas: princípios e aplicações. Piracicaba: Associação Brasileira para Pesquisa da Potassa e do Fosfato (Potafos) (1997).
7. Empresa Brasileira de Pesquisa Agropecuária (Embrapa). Manual de métodos de análises de solo (2° ed.) Rio de Janeiro, Brazil ISBN 85-85864-03-6 (1997).
8. Alvarez, V., Novais, V.H., Dias, R.F., Oliveira, L.E. Determinação e uso do fósforo remanescente. Boletim Informativo da Sociedade Brasileira de Ciência do Solo, 25, 27-32 (2000).
9. Quaggio, J.A. & Raji, B. V. Comparação de métodos rápidos para a determinação da matéria orgânica em solos. R. Bras. Ci. Solo, 3,184-187 (1979).
10. IEA / SP www.iea.agricultura.sp.gov.br (2018)
11. ANNT www.antt.gov.br (2018)
